# Effect of sutureless scleral fixed intraocular lens implantation on aphakic eyes: a system review and meta-analysis

**DOI:** 10.1186/s12886-023-03223-6

**Published:** 2023-12-06

**Authors:** Zhao Liu, Qian Xie, XingWang Chen, Bing Xie, ShanJun Cai

**Affiliations:** 1 Department of Ophthalmology, Affiliated Hospital of Zunyi Medical University, 149 Dalian Road, Zunyi, 563003 China; 2 Guizhou Eye Hospital, Zunyi, 563003 China; 3 Guizhou Provincial Branch of National Eye Disease Clinical Research Center, Zunyi, 563003 China; 4 Special Key Laboratory of Ocular Diseases of Guizhou Province, Zunyi Medical University, Zunyi, 563003 China

**Keywords:** Aphakia, Sutureless, SF-IOL, Efficacy, Meta

## Abstract

**Background:**

Sutureless scleral fixed intraocular lens implantation (SF-IOL) has become one of the mainstream schemes in clinical treatment of aphakic eyes because of its advantages, such as avoiding dislocation of intraocular lens or subluxation caused by suture degradation or fracture and significant improvement of postoperative visual acuity. However, a consensus on the relative effectiveness and safety of this operation and other methods is still lacking. This study aimed to compare the efficacy and safety of sutureless SF-IOL with other methods. Aphakia means that the lens leaves the normal position and loses its original function, including absence or complete dislocation and subluxation of the lens which could cause anisometropic amblyopia, strabismus, and loss of binocular function in children and adolescents. For adults, the loss of the lens could lead to high hyperopia and affect vision. Above all this disease can seriously affect the quality of life of patients.

**Methods:**

Literature about sutureless SF-IOL in PubMed, Cochrane Library, Embase, Web of Science, China National Knowledge Infrastructure, China Technical Journal VIP database, and Wanfang database published from 2000 to 2022 was reviewed. The weighted average difference was calculated by RevMan5.3 software for analysis. Two researchers independently selected the study and used the Cochrane collaboration tool to assess the risk of errors. Cochrane bias risk tool was used to evaluate the quality of evidence. This study is registered on PROSPERO (CRD42022363282).

**Results:**

The postoperative IOL-related astigmatism of sutureless SF-IOL was lower than that of suture SF-IOL, and there was statistical difference when we compared the absolute postoperative spherical equivalent after sutureless SF-IOL and suture SF-IOL. Indicating that the degree of refractive error after sutureless SF-IOL was lower. Meanwhile, the operation time of sutureless SF-IOL was shorter than that of suture SF-IOL. The subgroup analysis showed that the absolute postoperative spherical equivalent and astigmatism values in Yamane technique were lower than those in suture SF-IOL.

**Conclusion:**

Sutureless SF-IOL has the advantages of stable refraction, short operation time, and less postoperative complications. However, high-quality literature to compare these technologies is lacking. Some long-term follow-up longitudinal prospective studies are needed to confirm the findings.

**Supplementary Information:**

The online version contains supplementary material available at 10.1186/s12886-023-03223-6.

## Introduction

Aphakia means that the lens leaves the normal position and loses its original function, including absence or complete dislocation and subluxation of the lens [1]. This disease could cause anisometropic amblyopia, strabismus, and loss of binocular function in children and adolescents. For adults, the loss of the lens could lead to high hyperopia and affect vision. Aphakia has many causes, such as cataract surgery for lens posterior capsule injury (60%–75%); exogenous factors, such as lens injury during vitreoretinal surgery (8%–15%); and endogenous factors, such as Marfan syndrome, pseudo exfoliation syndrome, and idiopathic lens dislocation (15%–30%) [2–5].

At present, surgery is generally chosen in the clinical treatment of aphakic eyes. The conventional surgical procedures are as follows: anterior chamber intraocular lens implantation (AC-IOL), iris fixed intraocular lens implantation (IF-IOL), and scleral fixed intraocular lens implantation (SF-IOL). SF-IOL is divided into suture SF-IOL, glued SF-IOL, and sutureless SF-IOL. AC-IOL has been rarely used in clinic because it may lead to chamber angle injury, corneal endothelial injury, and even irreversible corneal endothelial decompensation. IF-IOL could be fixed by suture or without suture. Iris claw intraocular lens implantation, a kind of seamless intraocular lens, places the polymethyl methacrylate intraocular lens on the anterior surface of the iris and wraps the tactile device in the non-vascular part of the iris far away from the corneal endothelium and the iris angle [6]. The methods of suture and fixation include McCannel suture technique and Siepser sliding conjunctival suture technique [7, 8]. At present, this method still has good safety, and it is widely used in clinic. Suture SF-IOL is sutured to the sclera with 10–0 polypropylene or 9–0 polypropylene or expanded polytetrafluoroethylene suture [9]. Although suture fixation may induce complications, such as suture fracture and suture erosion [10, 11], with the continuous progress of science and technology, the procedure of this operation is constantly simplified and the injury is decreasing, hence the operation method that many clinical scholars are willing to choose. Glued SF-IOL effectively avoids the possible complications caused by suture by using fibrin glue to fix the IOL loop under the scleral flap or in the scleral tunnel. However, due to the high cost of fibrin glue and possible postoperative prion-related infection [12, 13], the scope of application of this operation is limited. Compared with the previously described surgery, sutureless SF-IOL greatly reduces tissue injury, reduces the incidence of postoperative complications, and shortens the postoperative recovery time by implanting the IOL loop into an artificially established scleral tunnel because it does not require suture, adhesion, and scleral cauterization [14]. In view of the above advantages, sutureless SF-IOL has become the mainstream surgical scheme for the clinical treatment of aphakic eyes.

However, no study has comprehensively compared the efficacy and safety of sutureless SF-IOL with suture SF-IOL. The present paper aimed to compare the relationship among different surgical methods and operation times, postoperative visual acuities, postoperative refractive states, postoperative intraocular lens inclinations, and postoperative complications to provide reference for follow-up clinical research.

## Materials and methods

Literature about sutureless SF-IOL in PubMed, Cochrane Library, Embase, Web of Science, China National Knowledge Infrastructure (CNKI), China Technical Journal (VIP) database, and Wanfang database published from 2000 to 2022 was reviewed. The weighted average difference (WMD) was calculated by RevMan5.3 software for analysis. Two researchers independently selected the study and used the Cochrane collaboration tool to assess the risk of errors. Cochrane bias risk tool was used to evaluate the quality of evidence.

### Search strategy

The Preferred Reporting Items for Systematic Reviews and Meta-Analyses (PRISMA, 2020) search strategy flow chart and checklist were used as guides in identifying and selecting relevant studies in EMBASE, Web of Science, Cochrane Library, PubMed, CNKI, VIP database, and WanFang database. All the searches were performed before February 2023. The search strategy included the following words and phrases: “(flanged intrascleral fixation) OR (SFIOL) OR (sutureless intraocular lens) OR (suture-free intraocular lens) OR (transconjunctival sutureless intrascleral fixation) OR (SFIOL).” Study selection was restricted to English and Chinese languages.

### Study selection

The selection criteria for this meta-analysis are as follows: 1) All studies should be designed with randomized controlled trials (RCT) or Non-randomised studies of the effects of interventions (NRSI). 2) All studies should be designed with prospective studies or retrospective studies with raw data. 3) Aphakia eyes were fixed with posterior chamber intraocular lens without suture and with glue-free sclera. 4) The prognosis should be evaluated by postoperative visual acuity best corrected visual acuity (BCVA), corrected distance visual acuity (CDVA), uncorrected visual acuity (UCVA), postoperative refractive status (myopia, hyperopia, and astigmatism), or intraocular lens inclination (°). The exclusion criteria are as follows: 1) review or meta-analysis articles; 2) no suture-free scleral fixation of posterior chamber intraocular lens; 3) reports of repetitive data articles; 4) postoperative visual acuity (BCVA, CDVA, and UCVA), absolute postoperative spherical equivalent, astigmatism(IOL-related astigmatism and surgery induce astigmatism), intraocular lens inclination (horizontal and vertical), refractive prediction error,or incidence of complications were not reported in the article.

### Data extraction

Two reviewers (Zhao Liu and Bing Xie) independently extracted data from each article. First, numerical data from tables, text, or figures were extracted. If these were not reported, data from graphs were extracted using a digital ruler software. In case data were not reported or unclear, authors were contacted by e-mail (maximum of two attempts). In case an outcome was measured at multiple timepoints, the data from the timepoint where efficacy was the highest were included. The following study identifiers were collected: title, author, design, number of eyes, country of origin, and publication year. At baseline, cohort age and sex distribution were collected. Outcomes were analyzed at final follow-up and included CDVA, BCVA, UCVA (Logmar Visual Acuity), postoperative visual acuity change, operation time, intraocular lens inclination (horizontal and vertical), absolute postoperative spherical equivalent, astigmatism (IOL-related astigmatism and surgery induce astigmatism), refractive prediction error, and incidence of complications. Logmar visual acuity was selected for analysis in this study.

### Methodological quality and assessment of studies

Cochrane Handbook for Systematic Reviews of Interventions: Several aspects of the included studies were assessed by the two researchers: random sequence generation, allocation concealment, blinding of participants and personnel, blinding of outcome assessment, incomplete outcome data, selective reporting, and other biases. A level of “high,” “low,” or “unclear” was given for each item [15].

### Data analysis

RevMan 5.3 was used to analyze the data and generate forest and funnel plots. The pooled estimate was reported as weighted mean differences (WMDs) with 95% confidence intervals (Cis) for continuous outcomes. Either the fixed-effect or random-effect model was used to pool the effect sizes. If I^2^ < 50% and *p* ≥ 0.1, the pooled outcomes were calculated by the fixed-effect model; otherwise, the random-effect model was applied. Stata 15.1 software was used to check publication bias, which was assessed using the funnel plots and Egger and Begg’s tests. Heterogeneity tests, including Q and I^2^ statistics, were calculated; 25%, 50%, and 75% I^2^ scores were considered low, moderate, and high heterogeneities, respectively. Subgroup analysis was categorized in accordance with surgical method.

## Results

### Study selection and characteristics

**Fig. 1:**
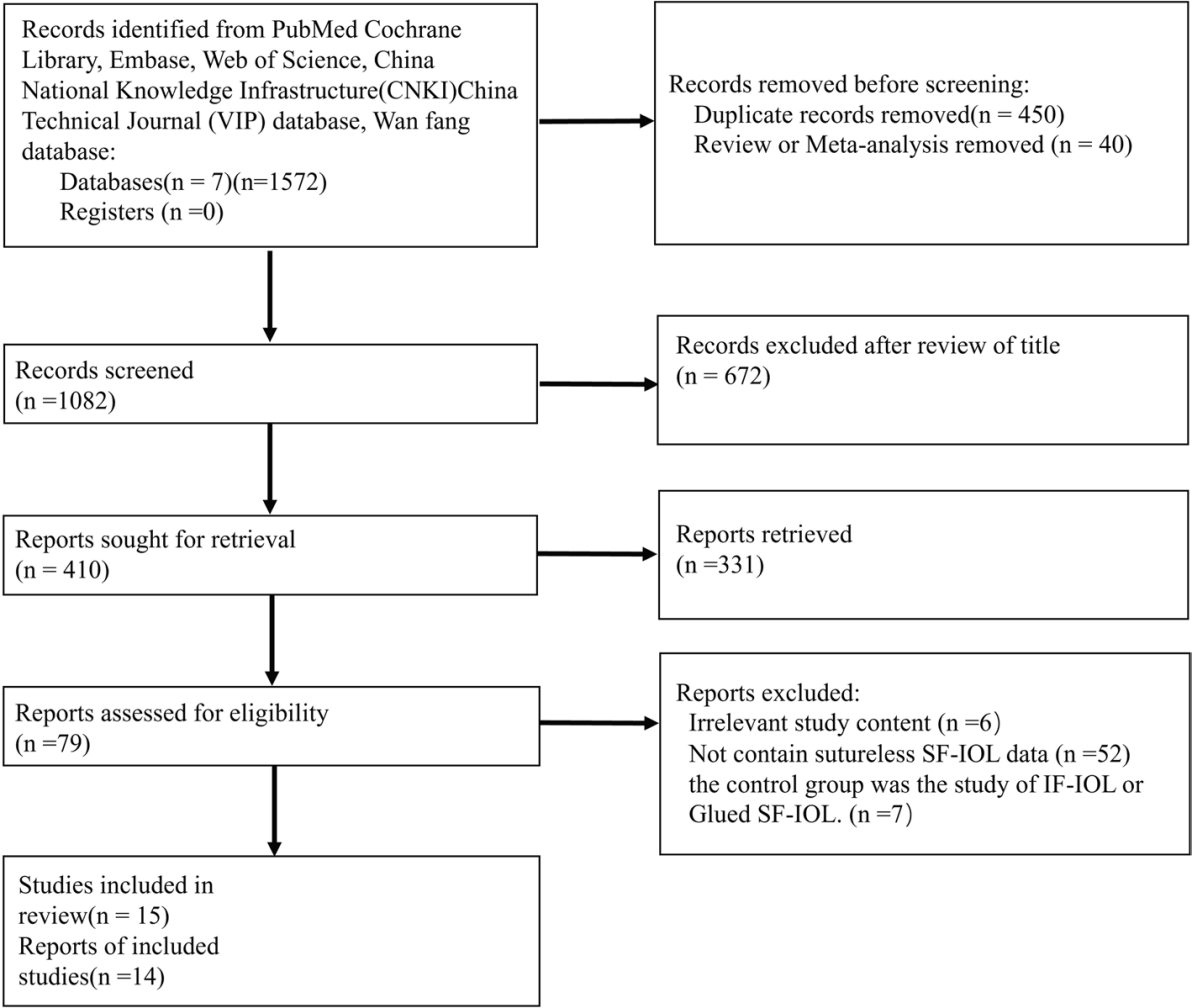
Flow diagram of selection studies

In this study, 470, 32, 527, 479, 21, 10, and 33 articles were retrieved from PubMed, Cochrane, Embase, Web of Science, CNKI, VIP, and Wanfang database, respectively, with a total of 1572 articles. Finally, 14 articles (including 15 studies) met the inclusion criteria, including one in Chinese and 13 in English. The selected scheme for literature retrieval and research is shown in Fig. 1.

Of the 1572 articles, 450 repetitive articles and 40 reviews/meta-analyses were deleted. After the title and abstract were screened, 672 articles were excluded after review of title. After reports sought for retrieval, 331 articles that were not related to sutureless SF-IOL were excluded. Finally, 79 articles were obtained for further research. A total of 65 articles were excluded as follows: six articles not related to sutureless SF-IOL,52 articles without control group and without sutureless SF-IOL and 7 articles that the control group was the study of IF-IOL or Glued SF-IOL. The earliest inclusion study was published in 2013. These articles are distributed in 10 countries: India, Turkey, South Korea, Poland, Japan, Germany, Brazil, Thailand, the United States, and China.Because the Daniel.et al.2021 [16] provided two control groups: one using Gore-Tex sutures and the other using Prolene sutures, this article provides two comparative studies.

Overall, 15 studies were included (14 articles), of which 13 were retrospective studies and two were prospective studies. A total of 817 eyes were examined in these 15 studies. Among them, 403 eyes underwent sutureless SF-IOL, 414 eyes underwent suture SF-IOL. A total of 420 (63.2%) and 244 (36.8%) men and women were included in the 14 studies, respectively. The average ages of patients who underwent sutureless SF-IOL and suture SF-IOL were 55.68 (4.80–76.04 years), 56.24 (14.33–72.87 years), respectively (Table 1). The surgical procedures and suture materials used in each study are listed in Table 2. The inclusion criteria and exclusion criteria of all studies are listed in Table 3. The average follow-up times of patients who underwent sutureless SF-IOL and suture SF-IOL were 11.84 (1.00–37.86 months) and 10.50 (1.00–17.28 months), respectively (Table 4).

**Table 1:** Basic information of all researches

Research	Male/female	Age of experimental group (Year,Mean ± SD)	Age of control group (Year,Mean ± SD)	Overall age (Year,Mean ± SD)
Gurkan.et al.2021 [30]	19/2	12 ± 7.14	14.33 ± 11.1-	12.3 ± 8.7
Jae.et al.2020 [20]	58/45	64.1 ± 6.9	63.3 ± 7.0	-
Gurkan.et al.2016 [9]	53/36	-	-	65.6 ± 12.2
Dariusz.et al.2016 [31]	27/15	-	-	53.5 ± 21.5
Kyu.et al.2021 [18]	57/13	62.92 ± 9.91	60.68 ± 12.92	61.49 ± 11.89
Yu.et al.2018 [23]	57/30	68.00 ± 16.05	68.73 ± 11.74	-
Manavi.et al.2016 [17]	63/46	55.03 ± 17.5	55.50 ± 18.3	55.2 ± 17.8
Mariya.et al.2022 [32]	15/15	-	-	68 ± 19.6
Yalcinbayir.et al.2021 [33]	49/25	61.6 ± 19.2	53.9 ± 19.2	-
Sül.et al.2020 [34]	30/8	76.04 ± 13.23	72.87 ± 15.28	-
Daniel.et al.2021(1) [16]	34/17	68.4 ± 3.3	58.7 ± 5.6	63.5 ± 2.6
Daniel.et al.2021(2) [16]	34/17	68.4 ± 3.3	61.4 ± 5.1	63.5 ± 2.6
Yodpong.et al.2018 [22]	22/26	62.5 ± 11.27	62.0 ± 9.36	-
Bruna.et al.2019 [19]	10/11	65.18 ± 12.65	62.6 ± 21.33	-
Zhang.et al.2021 [21]	18/8	50.5 ± 15.9	40.9 ± 14.7	-

**Table 2:** Summary of operation methods, suture type and scope of vitrectomy

Research	Operation method of experimental group	Operation method of control group	Suture type	Scope of vitrectomy
Gurkan.et al.2021 [30]	Gabor + vitrectomy	Suture fixation + vitrectomy	10–0 Polypropylene	PPV/Anterior
Jae.et al.2020 [20]	Yamane + 25Gvitrectomy	Suture fixation + 23Gvitrectomy	10–0 Polypropylene	-
Gurkan.et al.2016 [9]	Gabor + vitrectomy	Suture fixation + vitrectomy	10–0 Polypropylene	-
Dariusz.et al.2016 [31]	Gabor + vitrectomy	Suture fixation + vitrectomy	10–0 Polypropylene	Anterior
Kyu.et al.2021 [18]	Yamane + 25Gvitrectomy	Suture fixation + 25Gvitrectomy	10–0 Polypropylene	PPV
Yu.et al.2018 [23]	Yamane + 25Gvitrectomy	Suture fixation + 25Gvitrectomy	10–0 Polypropylene	-
Manavi.et al.2016 [17]	Gabor + 23/25Gvitrectomy	Suture fixation + 23/25Gvitrectomy	10–0 Polypropylene	PPV
Mariya.et al.2022 [32]	Yamane + vitrectomy	Suture fixation + vitrectomy	8–0 Gore-Tex	PPV
Yalcinbayir.et al.2021 [33]	Yamane + 23Gvitrectomy	Suture fixation + 23Gvitrectomy	10–0 Polypropylene	PPV
Sül.et al.2020 [34]	Yamane + vitrectomy	Suture fixation + vitrectomy	9–0 polypropylene	Anterior
Daniel.et al.2021(1) [16]	Yamane + 25Gvitrectomy	Suture fixation + 25Gvitrectomy	CV-8(7–0) Gore-Tex	Anterior
Daniel.et al.2021(2) [16]	Yamane + 25Gvitrectomy	Suture fixation + 25Gvitrectomy	10–0/9–0 Polypropylene	Anterior
Yodpong.et al.2018 [22]	Gabor + vitrectomy	Suture fixation + vitrectomy	10–0 Polypropylene	PPV /Anterior
Bruna.et al.2019 [19]	Gabor	Suture fixation	10–0 Polypropylene	-
Zhang.et al.2021 [21]	Yamane + vitrectomy	Suture fixation + vitrectomy	-	PPV /Anterior

*PS*: *PPV* Pars plana vitrectomy

**Table 3:** Inclusion criteria and exclusion criteria of all researches

Research	Inclusion criteria	Exclusion criteria
Gurkan.et al.2021 [30]	1. No improvement in visual function after eyeglasses or contact lens application. 2. Due to excessive irregular astigmatism, advanced crystalline lens decentration in which the edge of the crystalline lens came up to the optical axis. 3. The dislocation of the crystalline lens resulting in aphakia and secondary glaucoma due to lens dislocation 4. Patients who had undergone lens extraction with secondary IOL implantation at the same session	1. Glaucoma, ocular inflammation uveitis 2. Vitreoretinal interface disorder. 3. Preoperative retinal break with or without retinal detachment (RD)
Jae.et al.2020 [20]	1.IOL removal 2.IOL SF due to IOL dislocation	1. Any other retinal or choroidal diseases including diabetic retinopathy, retinal vein occlusion, retinal detachment, and age-related macular degeneration 2. A history of trauma including corneoscleral laceration 3. Corneal diseases including corneal opacity or corneal dystrophy. 4. Preoperative best-corrected visual acuity (BCVA) of less than 6/60 with aphakic correction
Gurkan.et al.2016 [9]	Vitrectomized aphakic eyes lacking capsular support	1. Follow-up period of < 3 months 2. Preoperative corneal opacity
Dariusz.et al.2016 [31]	1. Total absence of capsular bag, 2. History of eye trauma or complicated cataract surgery causing aphakia, 3. Regular 1 year follow-up	-
Kyu.et al.2021 [18]	1. Aphakia as a complication of cataract surgery with loss of capsular and/or zonular support 2. Posttraumatic aphakia 3. IOL dislocation without adequate capsular support 4. Congenital crystalline lens dislocation	-
Yu.et al.2018 [23]	1. IOL subluxation 2. Iris capture 3. Lens subluxation 4. Aphakia after cataract extraction	1. Patients with incomplete operative or postoperative medical records 2. Postoperative follow-up of < 1 month
Manavi.et al.2016 [17]	1. Post-traumatic aphakia 2. Aphakia as a complication of cataract surgery with loss of capsular and/or zonular support 3. Preoperative CDVA of at least 6/60 with aphakic correction 4. Dislocation of the crystalline lens after closed-globe injury 5. Aphakia after open-globe repair 6. Eyes that had additional surgical procedures, such as retinal detachment (RD) repair or vitrectomy for traumatic endophthalmitis, before scleral-fixated IOL implantation	-
Mariya.et al.2022 [32]	1. Aphakia with defcient capsular support 2. Dislocated and subluxated IOL or crystalline lens 3. Scleral fxation of IOL in combination with glaucoma drainage valve implant 4. Corneal Descemet’s stripping automated endothelial keratoplasty (DSAEK)	Less than 1 month follow up
Yalcinbayir.et al.2021 [33]	1. Partial or complete dislocation of the crystalline lens or IOL 2. Minimum follow up of 3 months	1. Adequate capsular support 2. History of ocular inflammation, 3. Bleeding disorders 4. Less than 3 months of follow-up
Sül.et al.2020 [34]	Aphakias after only cataract surgeries	1. Dilated pupil diameter > 4.5 mm 2. History of ocular disorders such as uveitis, high refractive error, retinitis pigmentosa, aphakic glaucoma 3. History of ocular trauma before cataract surgery
Daniel.et al.2021(1) [16]	1. Trauma 2. Previous (complicated) eye surgery 3. Zonular weakness (e.g. PEX, Marfan syndrome) 4. Congenital aniridia 5. Uveitis 6. Pigment dispersion glaucoma 7. Aphakia (e.g. after extraction of congenital cataract) 8. IOL refixation following previous scleral fixation	-
Daniel.et al.2021(2) [16]	1.Trauma 2.Previous (complicated) eye surgery 3.Zonular weakness (e.g. PEX, Marfan syndrome) 4.Congenital aniridia 5.Uveitis 6.Pigment dispersion glaucoma 7.Aphakia (e.g. after extraction of congenital cataract) 8.IOL refixation following previous scleral fixation	-
Yodpong.et al.2018 [22]	1. Aphakic with inadequate anterior capsular support 2. Phakic patients with severe zonular instability resulting from complicated cataract surgery, trauma 3. Prior retinal surgery	1. Severe retinal damage resulting in poor prognosis for visual benefit after surgery 2. Systemic disease whose anti-platelet therapy could not be discontinued
Bruna.et al.2019 [19]	1. Complication during phaco-emulsifcation 2. Lens subluxation and ocular trauma	3. Postoperative period shorter than 1 month 4. Uncooperative patients 5. Aniridia, ocular trauma with relevant disruption of the anterior segment anatomy 6. Previous glaucoma or corneal surgery (except for refractive surgery) 7. patients who did not agree to participate in the study and did not provide informed consent
Zhang.et al.2021 [21]	1. Trauma or other factors cause lesions of the suspensory ligament or injury of the lens capsule, resulting in insufficient strength to support the intraocular lens after extraction of the diseased lens 2. Postoperative aphakic eyes or capsule retention is not enough to support intraocular lens 3. Dislocation of intraocular lens can not be reset	1. Obvious scleral scar near the horizontal bilateral limbal 1 ~ 3 mm caused by eyeball penetrating injury or previous operation history 2. Patients with a history of scleritis, or significantly lengthened eye axis and thinned sclera in high myopia 3. The number of corneal endothelial cells is lower than 1000个·mm^−2^ 4. Preoperative intraocular pressure ≥ 21 mmHg (1 kPa = 7.5 mmHg) 5. Patients with active inflammation and fundus disease

**Table 4:** Summary of follow-up in all researches

Research	End time of follow-up (month)	Overall follow-up time (Mean ± SD, month)	Follow-up time of experimental group (Mean ± SD, month)	Follow-up time of control group (Mean ± SD, month)
Gurkan.et al.2021 [30]	-	-	16.40 ± 9.16	10 ± 5.7
Jae.et al.2020 [20]	12	-	-	-
Gurkan.et al.2016 [9]	-	7.0 ± 03.7	-	-
Dariusz.et al.2016 [31]	-	14.5 ± 2.2	-	-
Kyu.et al.2021 [18]	6	-	-	-
Yu.et al.2018 [23]	1	-	-	-
Manavi.et al.2016 [17]	-	-	20.42 ± 8.7	17.28 ± 8.6
Mariya.et al.2022 [32]	-	23 ± 15.2	-	-
Yalcinbayir.et al.2021 [33]		-	10m*(median)	11m*(median)
Sül.et al.2020 [34]	-	-	10.09 ± 2.75	9.93 ± 2.64
Daniel.et al.2021(1) [16]	12	-	-	-
Daniel.et al.2021(2) [16]	12	-	-	-
Yodpong.et al.2018 [22]	12	-	-	-
Bruna.et al.2019 [19]	-	-	4.36 ± 3.23	4.3 ± 3.56
Zhang.et al.2021 [21]	6	-	-	-

*Ps*: The Data type of Sül.et al.2020 [30] was median

### Methodologies for the bias of selected studies

**Fig. 2:**
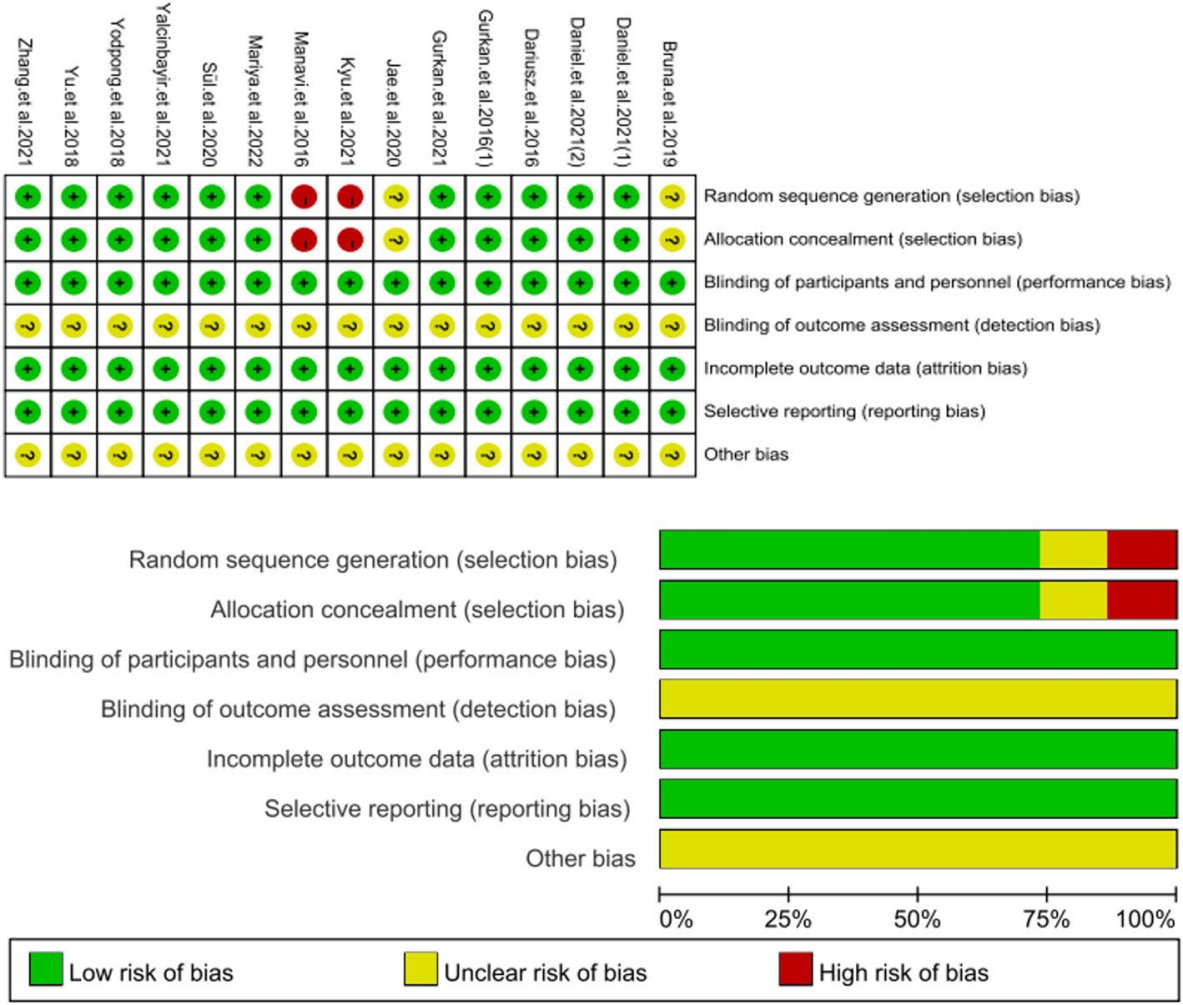
Risk of bias summary: review authors’ judgments about each risk of bias item for each included study

The quality of the included studies is shown in Fig. 2. Kyu.et al.2021 [17] and Manavi.et al.2016 [18] were listed as high risk because the choice of surgery was based on the surgeon's preference. Except for Bruna.et al.2019 [19] and Jae.et al.2020 [20], other studies clearly reported the use of random sequence generation. Due to the lack of information on these studies, assessing the hidden risk bias of allocation is not possible. Every medical worker does not inform patients about the treatment plans of other patients to protect the privacy of patients, so the risk of bias in the study is low. The results of the study were measured and registered by the researchers, but information was not sufficient to assess the risk bias of blind outcome assessments. No significant data loss or follow-up loss was reported in all studies, so the risk of bias is low. The reporting items in all the studies are complete, without missing reporting items and results, so the risk of reporting bias is low. Overall, no clear indication of other types of bias was observed.

### Data analysis

#### Visual acuity (Logmar Visual Acuity)

##### BCVA

**Fig. 3:**
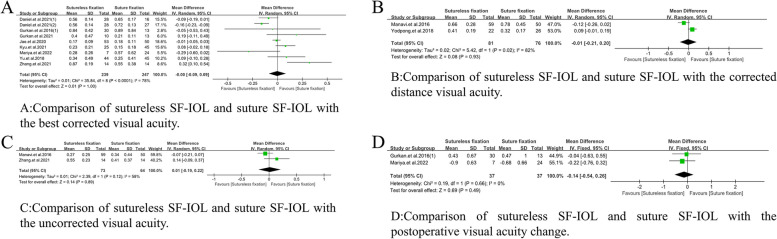
Meta-analysis on Postoperative Visual Acuity. **A** Comparison of sutureless SF-IOL and suture SF-IOL with the best corrected visual acuity. **B** Comparison of sutureless SF-IOL and suture SF-IOL with the corrected distance visual acuity. **C** Comparison of sutureless SF-IOL and suture SF-IOL with the uncorrected visual acuity. **D** Comparison of sutureless SF-IOL and suture SF-IOL with the postoperative visual acuity change

The BCVA after sutureless SF-IOL and suture SF-IOL were compared (WMD =  − 0.00, 95%CI = [− 0.09,0.09], *P* < 0.0001, I^2^ = 78%). No significant difference was found in the postoperative BCVA between the two intervention methods (Fig. 3A).

##### CDVA

The CDVA after sutureless SF-IOL and suture SF-IOL were compared (WMD =  − 0.01, 95% CI = [− 0.21,0.20], *P* = 0.02, I^2^ = 82%), without significant difference between the two groups (Fig. 3B).

##### UCVA

The UCVA after sutureless SF-IOL and suture SF-IOL were compared (WMD = 0.01, 95% CI = [− 0.19, 0.22], *P* = 0.12, I^2^ = 58%). No significant statistical difference was found after comparison (Fig. 3C).

##### Postoperative visual acuity change

The postoperative visual acuity changes after sutureless SF-IOL and suture SF-IOL were compared (WMD =  − 0.14, 95% CI = [− 0.54, 0.26], *P* = 0.66, I^2^ = 0%), without statistical difference between the two (Fig. 3D).

#### Operation time

**Fig. 4:**
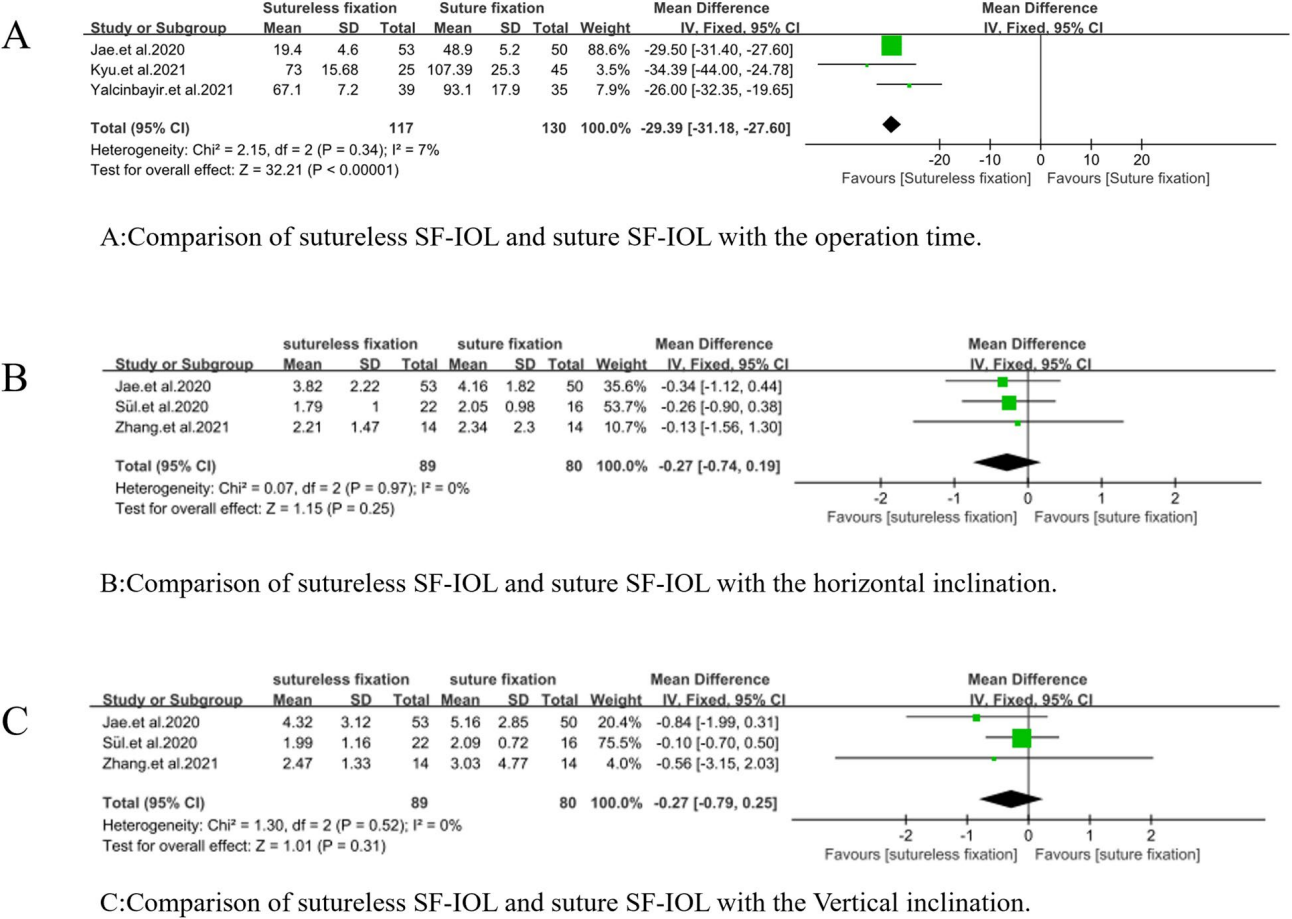
Meta-analysis on operation time and intraocular lens inclination. **A** Comparison of sutureless SF-IOL and suture SF-IOL with the operation time. **B** Comparison of sutureless SF-IOL and suture SF-IOL with the horizontal inclination. **C** Comparison of sutureless SF-IOL and suture SF-IOL with the Vertical inclination

The operation times of sutureless SF-IOL and suture SF-IOL were compared (WMD =  − 29.39, 95% CI = [− 31.18, − 27.60], *P* = 0.34, I^2^ = 7%). The difference between the two was statistically significant, and the time required for sutureless SF-IOL was shorter (Fig. 4A).

##### Intraocular Lens Inclination (horizontal and vertical)

The horizontal and vertical inclinations of intraocular lens after sutureless SF-IOL and suture SF-IOL were compared. (WMD =  − 0.27, 95% CI = [− 0.74, 0.19], *P* = 0.97, I^2^ = 0%; WMD =  − 0.27, 95% CI = [− 0.79, 0.25], *P* = 0.52, I^2^ = 0%). No significant difference was observed in the IOL inclination between the two interventions (Fig. 4B and C).

##### Absolute postoperative spherical equivalent

**Fig. 5:**
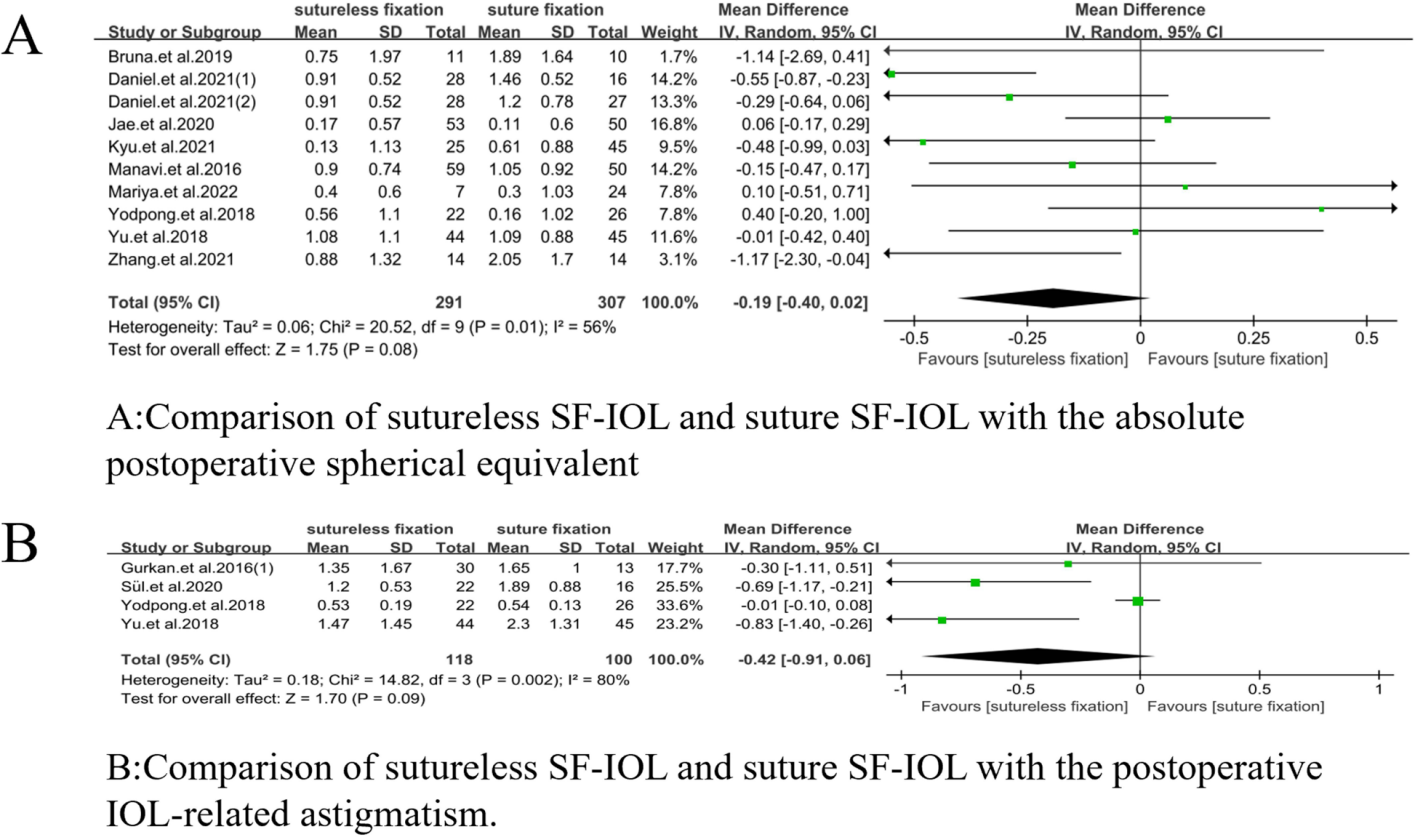
Meta-analysis on absolute postoperative spherical equivalent, astigmatism and refractive prediction error. **A** Comparison of sutureless SF-IOL and suture SF-IOL with the absolute postoperative spherical equivalent. **B** Comparison of sutureless SF-IOL and suture SF-IOL with the postoperative IOL-related astigmatism

The absolute postoperative spherical equivalent of sutureless SF-IOL and suture SF-IOL at the last follow-up was compared (WMD =  − 0.19, 95% CI = [− 0.40, 0.02], *P* = 0.01, I^2^ = 56%). No significant difference was observed between the two groups (Fig. 5A).

##### Postoperative astigmatism

The postoperative IOL-related astigmatism between sutureless SF-IOL and suture SF-IOL during the last follow-up was compared (WMD =  − 0.42, 95% CI = [− 0.91, 0.06], *P* = 0.002, I^2^ = 80%). no significant difference was found between the two (Fig. 5B).

#### Postoperative complications

##### Iris clamping

**Fig. 6:**
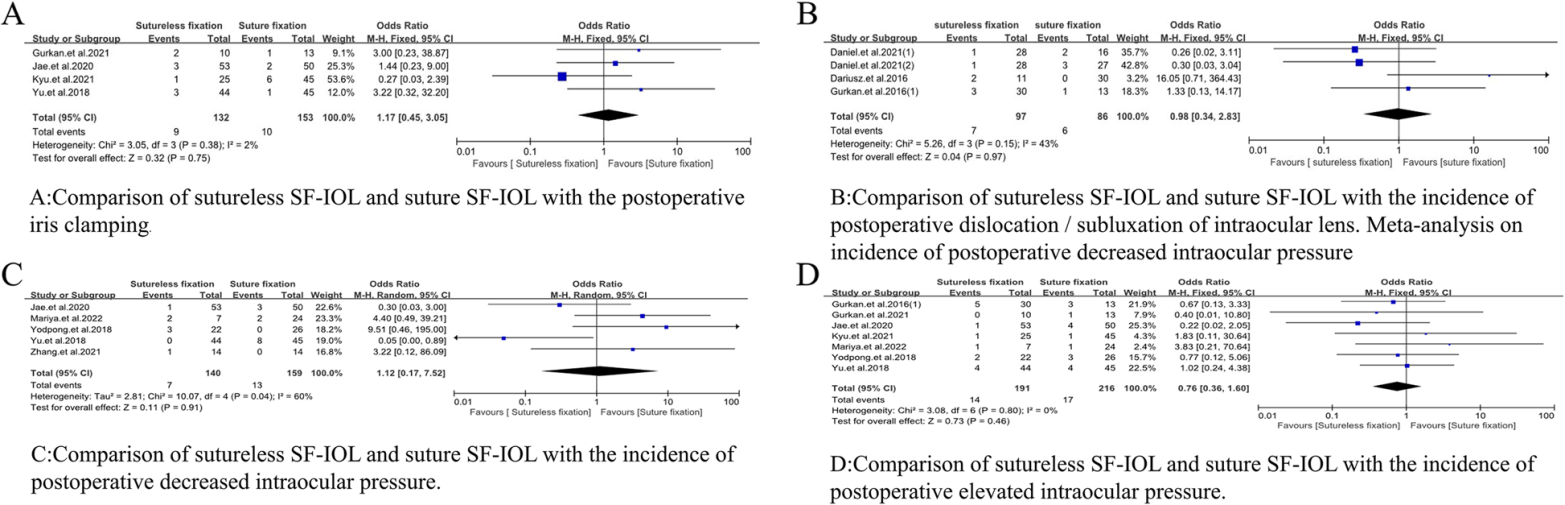
Meta-analysis on postoperative complications. **A** Comparison of sutureless SF-IOL and suture SF-IOL with the postoperative iris clamping. **B** Comparison of sutureless SF-IOL and suture SF-IOL with the incidence of postoperative dislocation / subluxation of intraocular lens. Meta-analysis on incidence of postoperative decreased intraocular pressure. **D** Comparison of sutureless SF-IOL and suture SF-IOL with the incidence of postoperative decreased intraocular pressure. **F** Comparison of sutureless SF-IOL and suture SF-IOL with the incidence of postoperative elevated intraocular pressure

The number of cases of iris clamping after sutureless SF-IOL and suture SF-IOL was compared (OR = 1.17, 95% CI = [0.45, 3.05], *P* = 0.38, I^2^ = 2%), and no significant difference was observed (Fig. 6A).

##### Dislocation/subluxation of intraocular lens

The number of cases of intraocular lens dislocation/subluxation after sutureless SF-IOL and suture SF-IOL was compared (OR = 0.98, 95% CI = [0.34, 2.83], *P* = 0.15, I^2^ = 43%). No significant difference was observed between the two interventions (Fig. 6B).

##### Decreased intraocular pressure

The incidences of intraocular pressure decrease after sutureless SF-IOL and suture SF-IOL were compared (OR = 1.12, 95% CI = [0.17, 7.52], *P* = 0.04, I^2^ = 60%), and no significant difference was found between the two groups (Fig. 6C).

##### Elevated intraocular pressure

The incidences of elevated intraocular pressure after sutureless SF-IOL and suture SF-IOL were compared (OR = 0.76, 95% CI = [0.36, 1.60], *P* = 0.80, I^2^ = 0%), without significant difference between the two groups (Fig. 6D).

### Research and analysis

In this paper, the funnel chart and sensitivity map were analyzed for the research with I^2^ ≥ 50% and the number of studies ≥ 5 after merger.

#### BCVA

**Fig. 7:**
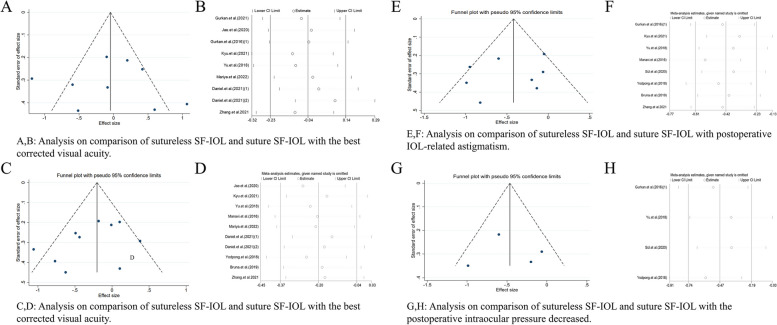
Funnel chart analysis and sensitivity map analysis on the best corrected visual acuity. **A** funnel chart analysis on comparison of sutureless SF-IOL and suture SF-IOL with the best corrected visual acuity. **B** sensitivity map analysis on comparison of sutureless SF-IOL and suture SF-IOL with the best corrected visual acuity. **C** funnel chart analysis on comparison of sutureless SF-IOL and suture SF-IOL with the best corrected visual acuity. **D** sensitivity map analysis on comparison of sutureless SF-IOL and suture SF-IOL with the best corrected visual acuity. **E** funnel chart analysis on comparison of sutureless SF-IOL and suture SF-IOL with the postoperative IOL-related astigmatism. **F** sensitivity map analysis on comparison of sutureless SF-IOL and suture SF-IOL with postoperative IOL-related astigmatism. **G** funnel chart analysis on comparison of sutureless SF-IOL and suture SF-IOL with the postoperative intraocular pressure decreased. **H** sensitivity map analysis on comparison of sutureless SF-IOL and suture SF-IOL with postoperative intraocular pressure decreased

A comparative study of sutureless SF-IOL and suture SF-IOL (Fig. 7A and B). The funnel chart analysis found that the distribution of the study was symmetrical, but two studies exceeded the CI.

#### Absolute postoperative spherical equivalent

A comparative study of sutureless SF-IOL and suture SF-IOL (Fig. 7C and D). The funnel chart found that the distribution of the study was symmetrical, but one study exceeded the CI.

#### Postoperative IOL-related astigmatism

A comparative study of sutureless SF-IOL and suture SF-IOL (Fig. 7E and F). The funnel chart analysis found that the distribution of the study was symmetrical, and no research was beyond the CI.

#### Postoperative intraocular pressure decreased

A comparative study of sutureless SF-IOL and suture SF-IOL (Fig. 7G and H). The funnel chart analysis found that the distribution of the study was symmetrical, but one study exceeded the CI. The sensitivity map analysis also found that one study had a great effect on the analysis of the results.

## Discussion

In this systematic review and meta-analysis, one Chinese literature and 13 English articles were selected, of which 15 studies were included.

Combined with the study, no significant difference was found in the postoperative visual acuity (BCVA, CDVA and UCVA) between sutureless SF-IOL and suture SF-IOL. Meanwhile, the operation time of sutureless SF-IOL is shorter than that of suture SF-IOL.

The intraocular lens inclination (horizontal and vertical), absolute postoperative spherical equivalent, and astigmatism after sutureless SF-IOL were compared with those after suture SF-IOL. The difference was not statistically significant.

For the study of postoperative complications, the common postoperative complications, such as dislocation/subluxation of intraocular lens, iris clamping, pupil deformation, elevated pressure and decreased intraocular pressure were analyzed. No statistical difference was found in these comparisons.

**Fig. 8:**
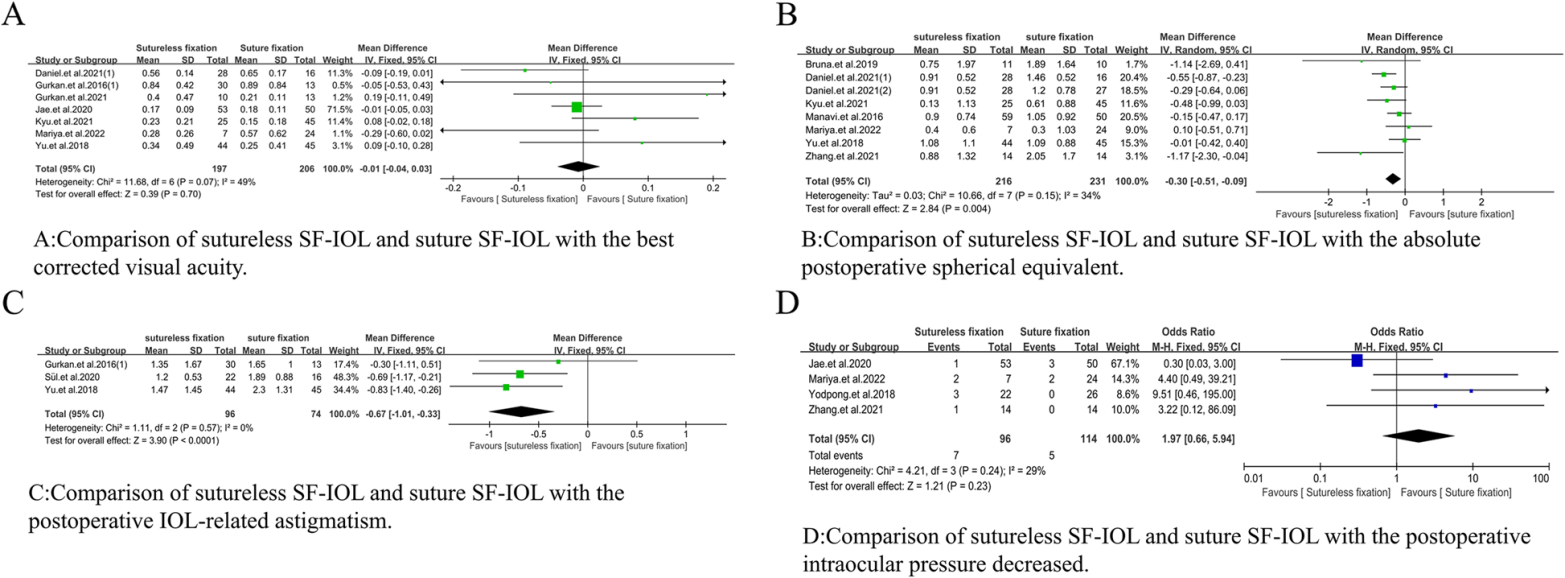
Meta-analysis on best corrected visual acuity,absolute postoperative spherical equivalent, postoperative IOL-related astigmatism and postoperative intraocular pressure decreased after exclude some researches. **A** Comparison of sutureless SF-IOL and suture SF-IOL with the best corrected visual acuity. **B** Comparison of sutureless SF-IOL and suture SF-IOL with the absolute postoperative spherical equivalent. **C** Comparison of sutureless SF-IOL and suture SF-IOL with the postoperative IOL-related astigmatism. **D** Comparison of sutureless SF-IOL and suture SF-IOL with the postoperative intraocular pressure decreased

In addition, a funnel chart and sensitivity map analysis were performed for the comparative studies with I^2^ ≥ 50% and the number of studies ≥ 5 after the merger. In accordance with the results, the relevant literature was reviewed to analyze the causes of heterogeneity. The causes of heterogeneity are described in Table 5. According to the analysis of funnel chart and sensitivity map, the main sources of heterogeneity in BCVA after sutureless SF-IOL and suture SF-IOL were the studies of Daniel.et al.2021(2) [16] and Zhang.et al.2021 [21]. After the studies of Daniel.et al.2021(2) [16] and Zhang.et al.2021 [21] were excluded, the heterogeneity decreased (WMD =  − 0.01, 95% CI = [− 0.04, 0.03], *p* = 0.07, I^2^ = 49%; Fig. 8A), which also proved the present work’s idea. In the comparison of absolute postoperative spherical equivalent between sutureless SF-IOL and suture SF-IOL, the main source of heterogeneity was the study of Yodpong.et al. 2018 [22] and Jae.et al.2020 [20]. After the study of Yodpong.et al. 2018 [22] and Jae.et al.2020 [20] was excluded, the heterogeneity decreased (WMD =  − 0.30, 95% CI = [-0.51, -0.09], *p* = 0.15, I^2^ = 34%; Fig. 8B). A significant difference was found in the refractive values between sutureless SF-IOL and suture SF-IOL, and the refractive value was lower after sutureless SF-IOL. In the comparison of the postoperative IOL-related astigmatism between sutureless SF-IOL and suture SF-IOL, the main sources of heterogeneity were the studies of Kyu.et al.2021 [18] and Yodpong.et al.2018 [22]. After the above two studies were excluded, a decrease in heterogeneity was found (WMD =  − 0.67, 95% CI = [− 1.01, − 0.33], *p* = 0.57, I^2^ = 0%; Fig. 8C), and A significant difference was observed between the two interventions. In the comparison of intraocular pressure reduction after sutureless SF-IOL and suture SF-IOL, the main source of heterogeneity was the study of Yu. etal.2018 [23]. After the study of Yu.et al.2018 [23] was excluded, a decrease in heterogeneity was found (OR = 1.97, 95% CI = [0.66, 5.94], *p* = 0.24, I^2^ = 29%; Fig. 8D).

**Table 5:** Cause analysis of heterogeneity

Study	Analysis	The reason for the heterogeneity
Daniel.et al.2021(2) [16]	Comparative study of the BCVA between sutureless SF-IOL and suture SF-IOL	10–0 polypropylene and 9–0 polypropylene were both used in this study
Zhang.et al.2021 [21]	Comparative study of the BCVA between sutureless SF-IOL and suture SF-IOL	The specific types of sutures were not mentioned, and the scope of vitrectomy included anterior and total vitreous
Yodpong.et al.2018 [22]	Comparative study ofthe Absolute Postoperative Spherical Equivalent between sutureless SF-IOL and suture SF-IOL	This study use a new technique which may lead the postoperative IOL-related astigmatism by induce the IOL tilt
Jae.et al. 2020 [20]	Comparative study ofthe Absolute Postoperative Spherical Equivalent between sutureless SF-IOL and suture SF-IOL	The caliber of vitrectomy for Yamane technique was 25G and that of suture scleral IOL implantation was 23G
Kyu.et al.2021 [18]	Comparative study ofthe postoperative IOL-related astigmatism between sutureless SF-IOL and suture SF-IOL	Remove the old IOL and insert a new IOL by a 2.75-mm corneal incision
Yodpong.et al.2018 [22]	Comparative study ofthe postoperative IOL-related astigmatism between sutureless SF-IOL and suture SF-IOL	This study use a new technique which may lead the postoperative IOL-related astigmatism by induce the IOL tilt
Yu. et al.2018 [23]	Comparative study ofthe intraocular pressure reduction between sutureless SF-IOL and suture SF-IOL	The suture fixation group had a larger incision and more operations than the non-suture fixation group in this study
Jae.et al. 2020 [20]	Comparative study ofthe absolute postoperative spherical equivalent between Yamane technique and suture SF-IOL	The caliber of vitrectomy for Yamane technique was 25G and that of suture scleral IOL implantation was 23G
Yodpong.et al. 2018 [22]	Comparative study ofthe absolute postoperative spherical equivalent between Gabor technique and suture SF-IOL	The forward movement of the optical part of the intraocular lens after the IOL haptic enters the scleral tunnel during IOL fixation

**Fig. 9:**
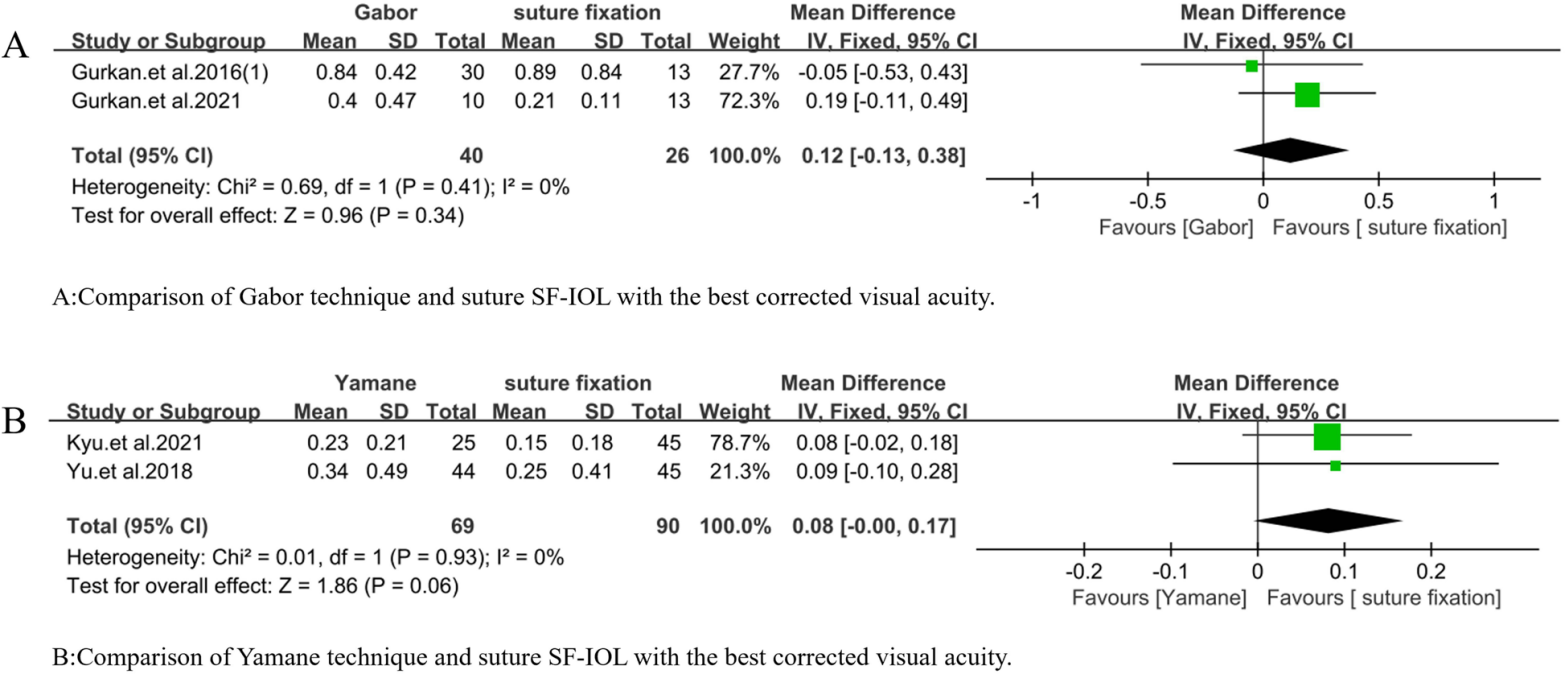
Meta-analysis on the best corrected visual acuity of subgroup techniques. **A** Comparison of Gabor technique and suture SF-IOL with the best corrected visual acuity. **B** Comparison of Yamane technique and suture SF-IOL with the best corrected visual acuity

**Fig. 10:**
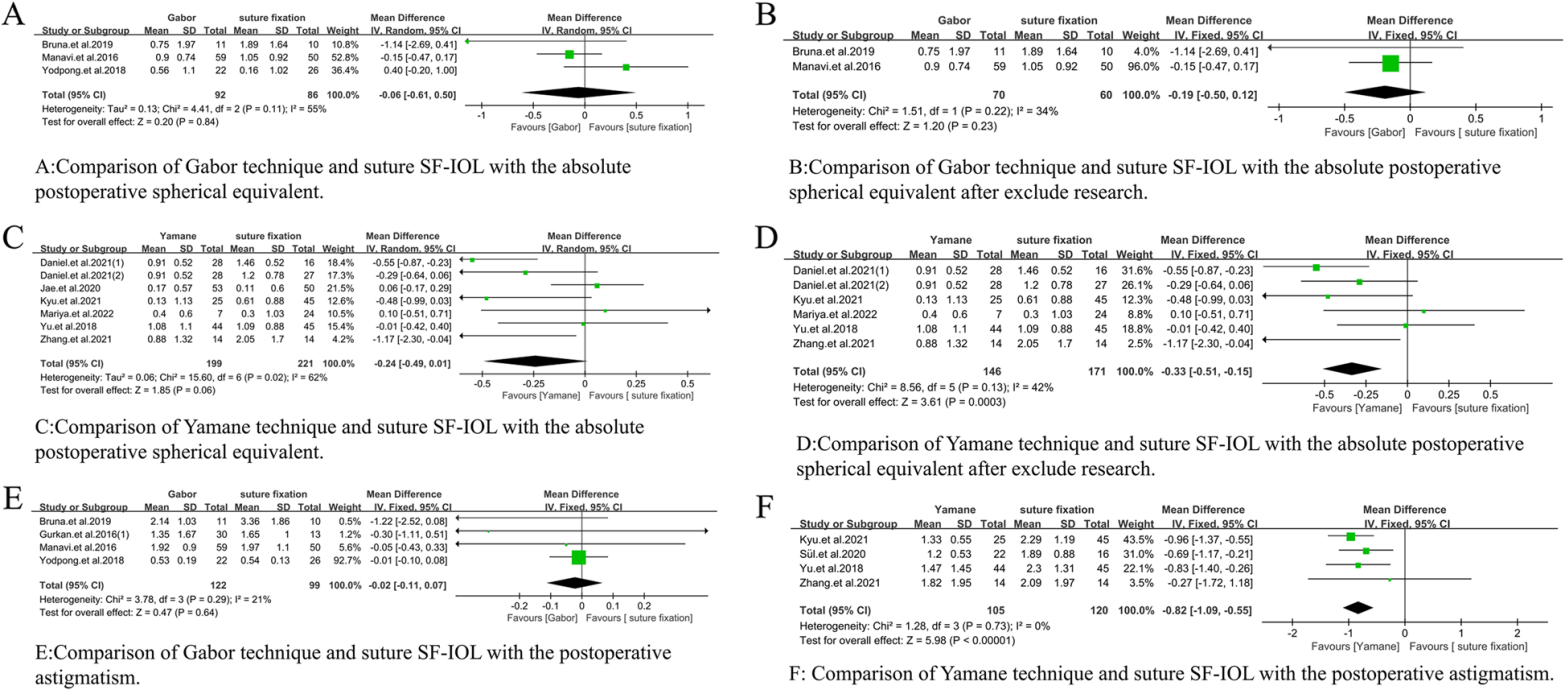
Meta-analysis on the absolute postoperative spherical equivalent and the postoperative astigmatism of subgroup techniques. **A** Comparison of Gabor technique and suture SF-IOL with the absolute postoperative spherical equivalent. **B** Comparison of Gabor technique and suture SF-IOL with the absolute postoperative spherical equivalent after exclude research. **C** Comparison of Yamane technique and suture SF-IOL with the absolute postoperative spherical equivalent. **D** Comparison of Yamane technique and suture SF-IOL with the absolute postoperative spherical equivalent after exclude research. **E** Comparison of Gabor technique and suture SF-IOL with the postoperative astigmatism. **F** Comparison of Yamane technique and suture SF-IOL with the postoperative astigmatism

In addition, the types of sutureless SF-IOL were divided into Gabor, Agarwal, and Yamane techniques. A subgroup analysis of the three techniques was conducted to determine the sources of heterogeneity in some of the comparative studies. In accordance with the number of studies, the difference in BCVA between Gabor technique and suture SF-IOL was compared (WMD = 0.12, 95% CI = [− 0.13, 0.38], *P* = 0.41, I^2^ = 0%; Fig. 9A). Although no statistical difference was found between the two, the heterogeneity of the study was low. In addition, the difference in BCVA between Yamane technique and suture SF-IOL was compared (WMD = 0.08, 95% CI = [− 0.00, 0.17], *P* = 0.93, I^2^ = 0%; Fig. 9B). No statistical difference was observed between the two, and the heterogeneity of the study was low. Meanwhile, the difference in the absolute postoperative spherical equivalent between Gabor technique and suture SF-IOL was compared (WMD =  − 0.06, 95% CI = [− 0.61, 0.50], *P* = 0.11, I^2^ = 55%; Fig. 10A). No statistical difference was found between them, but the heterogeneity was high. After the study of Yodpong.et al. 2018 [22] was analyzed and excluded, the heterogeneity decreased (WMD =  − 0.19, 95% CI = [− 0.50, 0.12], *p* = 0.22, I^2^ = 34%; Fig. 10B), and the difference was not statistically significant. In accordance with the original analysis, the heterogeneity caused by the study of Yodpong.et al. (2018) [22] is due to unnecessary refractive errors caused by the forward movement of the optical part of the intraocular lens after the IOL haptic enters the scleral tunnel during IOL fixation. The difference in the absolute postoperative spherical equivalent between Yamane technique and suture SF-IOL was also compared (WMD =  − 0.24, 95% CI = [− 0.49, 0.01], *P* = 0.02, I^2^ = 62%; Fig. 10C). No statistical difference was observed between them, but the heterogeneity was high. The study of Jae.et al. (2020) [20] was analyzed and excluded to further study and analyze the effect of reducing heterogeneity. The heterogeneity of the study decreased, and a statistical difference was found between the two (WMD =  − 0.33, 95% CI = [− 0.51, − 0.15], *P* = 0.13, I^2^ = 42%; Fig. 10D), which proved that the absolute postoperative spherical equivalent after Yamane technique was lower than that after suture SF-IOL. In accordance with the original analysis, the reason for the heterogeneity in the study of Jae.et al. (2020) [20] is that the caliber of vitrectomy for Yamane technique was 25G and that of suture scleral IOL implantation was 23G.The difference in astigmatism between Gabor technique and suture SF-IOL was also compared (WMD =  − 0.02, 95% CI = [− 0.11, 0.07], *P* = 0.29, I^2^ = 21%; Fig. 10E), without statistical difference between them. Meanwhile, a statistically significant difference in astigmatism was found between Yamane and suture SF-IOL (WMD =  − 0.82, 95% CI = [− 1.09, − 0.55], *P* = 0.73, I^2^ = 0%; Fig. 10F), which proved that the astigmatism in Yamane technique was smaller than that in the suture group.

To sum up, according to the analysis of the research results, the postoperative IOL-related astigmatism and absolute postoperative spherical equivalent of sutureless SF-IOL were lower than that of suture SF-IOL, indicating that the degree of refractive error after sutureless SF-IOL was lower. Meanwhile, the operation time of sutureless SF-IOL was shorter than that of suture SF-IOL. The subgroup analysis showed that the absolute postoperative spherical equivalent and astigmatism values in Yamane technique were lower than those in suture SF-IOL, with statistical value. This finding also proved that Yamane’s technique is superior to Gabor’s technique. Therefore, through the above analysis and summary, we believe that, compared with suture SF-IOL, Sutureless SF-IOL has the advantages of shorter operation time, stable postoperative refractive state and the incidence of postoperative complications. Yamane technique is superior to suture SF-IOL and Gabor’s technique in subgroup analysis.

In 1997, Italian scholars Ricardo Maggi and Carlo Maggi [24] put forward the concept of sutureless SF-IOL for the first time in view of the possible postoperative complications caused by suture. They chose an intraocular lens with three long 8.5 mm rings (made of polytetrafluoroethylene), which were fixed to the sclera through a special needle at 2:00, 6:00, and 10:00. This surgical method uses conjunctiva and sclera to cover the intraocular lens loop to avoid exposure to the outside of the eye, thus reducing the incidence of intraocular infection after operation. In addition, when using this method, if intraocular lens displacement or deviation occurs during operation, it could be corrected by adjusting the length of the ring. This method provides a new fixation method and fixed site for suture-free scleral interlamellar intraocular lens implantation. German scholar Gabor proposed seamless intraocular lens implantation in the ciliary sulcus in 2007 [25]. This method uses a standard three-piece foldable intraocular lens. The scleral tunnel is made by a common No. 24 cannula needle at the distance from the limbal of 1.5–2.0 mm, and then the IOL loop is brought into the scleral tunnel by tweezers, and the IOL loop is buried in the sclera. This method is the first time that the concept of “sutureless and glueless” has been applied to the clinic. During the follow-up period, no serious complications were noted, and in the later follow-up, 96.8% of the 63 patients had stable IOL. The work of Gabor provides a practical basis for the follow-up research, but the operation of the intraocular lens loop into the scleral tunnel is more difficult and takes a long time because of the equipment. In 2008, Agarwal [26] proposed to make a scleral flap to fix the IOL loop, which makes it more convenient to fix the IOL loop. Compared with the method proposed by Gabor, Agarwal expands the operating space by changing the shape of the sclera incision to make the IOL loop easier to draw out from the eye. However, excessive sclerotomy may cause postoperative scleral thinning and softening, resulting in other serious complications, and the technique closes the scleral flap with fibrin glue, which may lead to postoperative prion-associated infection [12]. In 2014, Yamane of Yokohama City University in Japan introduced a new technique at the annual meeting of ophthalmology in the United States [27]. This technique uses two No. 27 needles to perform scleral lamellar anatomy. It combines the advantages of Gabor and Akira’s methods, not only reducing the size of scleral incision and the probability of incision leakage and low intraocular pressure but also simplifying the operation procedure and shortening the operation time. Although some defects, such as difficulty in intraoperative operation and unstable intraocular lens fixation after operation [28], could still be noted, this method has been popularized after the report. In view of the poor stability of postoperative IOL, Yamane [29] proposed to use two No. 30 cannula needles to make a scleral tunnel and increase the fixation of IOL by cauterizing the end of IOL loop. The scleral damage of the 30 G needle used in this operation is less than that of the Nos. 25 and 27 G needle, and the smaller the diameter of the cannula needle is, the higher the stability of the scleral tunnel. No cases of dislocation were identified during the follow-up period in this study. So far, sutureless SF-IOL has been rapidly promoted.

At present, sutureless SF-IOL has become the mainstream surgical scheme for the clinical treatment of aphakic eyes, because it does not require suture, adhesion, scleral cauterization, and other operations. As a result, the tissue injury is greatly reduced, the incidence of postoperative complications is reduced, and the postoperative recovery time is shortened [14]. According to the comparative analysis of the present study, this method has the advantages of shorter operation time, more stable refractive state, and lower incidence of complications than suture SF-IOL.

The topic of comparing various secondary IOL techniques is a difficult and controversial issue, as well-designed comparative studies are rare and challenging to conduct due to the variations in each technique and the learning curve for each operator. The postoperative outcomes especially the operation time and visual acuity may be greatly influenced by the experience of the surgeon. Meanwhile, the reason of aphakia may influence the postoperative outcomes. Surgically, traumatically or congenitally induced aphakia may be accompanied with glaucoma, macular oedema and other ocular dysplasia. However, we believe that by comparing one surgical method with other surgical methods to find out the shortcomings of this surgical method and constantly adjust it is a feasible way to improve the quality of medical care. Besides, the main limitation of this study is the retrospective nature of most studies. The location of the IOL is determined by the surgeon’s preference and the patient’s eye history, leading to differences in baseline characteristics, which may affect the results between groups. Standardization among studies is also lacking, resulting in inconsistencies in clinical indications, surgical techniques, surgeon experience, reported results and limited duration in several studies. Meanwhile, duration of follow up is very important. The visual acuity, refractive errors, inclination of intraocular lens, and the postoperative complications may change with time. Thus, the quality of the evidence of the results differs, and it may lead to inconsistencies and statistical heterogeneity. In addition, the sample size of the study is small, which leads to the low statistical ability of some analyses. Due to the limited number of studies, this paper could not fully compare the differences among Yamane, Gabor, and Agarwal’s techniques.

## Conclusion

According to the analysis of forest map, sensitivity map and funnel map, we found that the operation time of sutureless SF-IOL was shorter than that of suture SF-IOL, there was statistical difference when we compared the IOL-related astigmatism and the absolute postoperative spherical equivalent after sutureless SF-IOL and suture SF-IOL. Yamane technique is superior to suture SF-IOL and Gabor’s technique in subgroup analysis. In summary, Sutureless SF-IOL has the advantages of stable refraction and short operation time. However, high-quality literature to compare these technologies is lacking. Some long-term follow-up longitudinal prospective studies are needed to confirm the findings.

### Supplementary Information

**Additional file 1. **Search strategy.

**Additional file 2. **PRISMA 2020 Checklist.

**Additional file 3. **PRISMA 2020 flow diagram.

## Data Availability

The datasets used and/or analyzed during the current study are available from the corresponding author on reasonable request.

## Abbreviations

AC-IOL: Anterior Chamber Intraocular Lens
BCVA: Best Corrected Visual Acuity
CDVA: Corrected Distance Visual Acuity
Glued SF-IOL: Glued Scleral Fixed Intraocular Lens
IF-IOL: Iris Fixed Intraocular Lens
SF-IOL: Scleral Fixed Intraocular Lens
Sutureless SF-IOL: Sutureless Scleral Fixed Intraocular Lens
Suture SF-IOL: Suture Scleral Fixed Intraocular Lens
UCVA: Uncorrected Visual Acuity
WMDs: Weighted Mean Differences
CIS: Confidence Intervals
VH: Vitreous Hemorrhage
CME: Cystoid Macular Edema
