# Supplementary material for: Effect of sutureless scleral fixed intraocular lens implantation on aphakic eyes: a system review and meta-analysis
Source: BMC Ophthalmol. 2023 Dec 6;23:493. doi: 10.1186/s12886-023-03223-6 (PMC10698919; doi:10.1186/s12886-023-03223-6)
Supplement: Supplementary file 1 — Additional file 1. Search strategy. [file 12886_2023_3223_MOESM1_ESM.pdf]

PUBMED：

(suture–free intraocular lens) OR (SFIOL) OR (sutureless intraocular lens) OR (flanged intrascleral fixation) OR (transconjunctival sutureless intrascleral fixation) OR (SFIOL) Filters: from 2000 – 2022

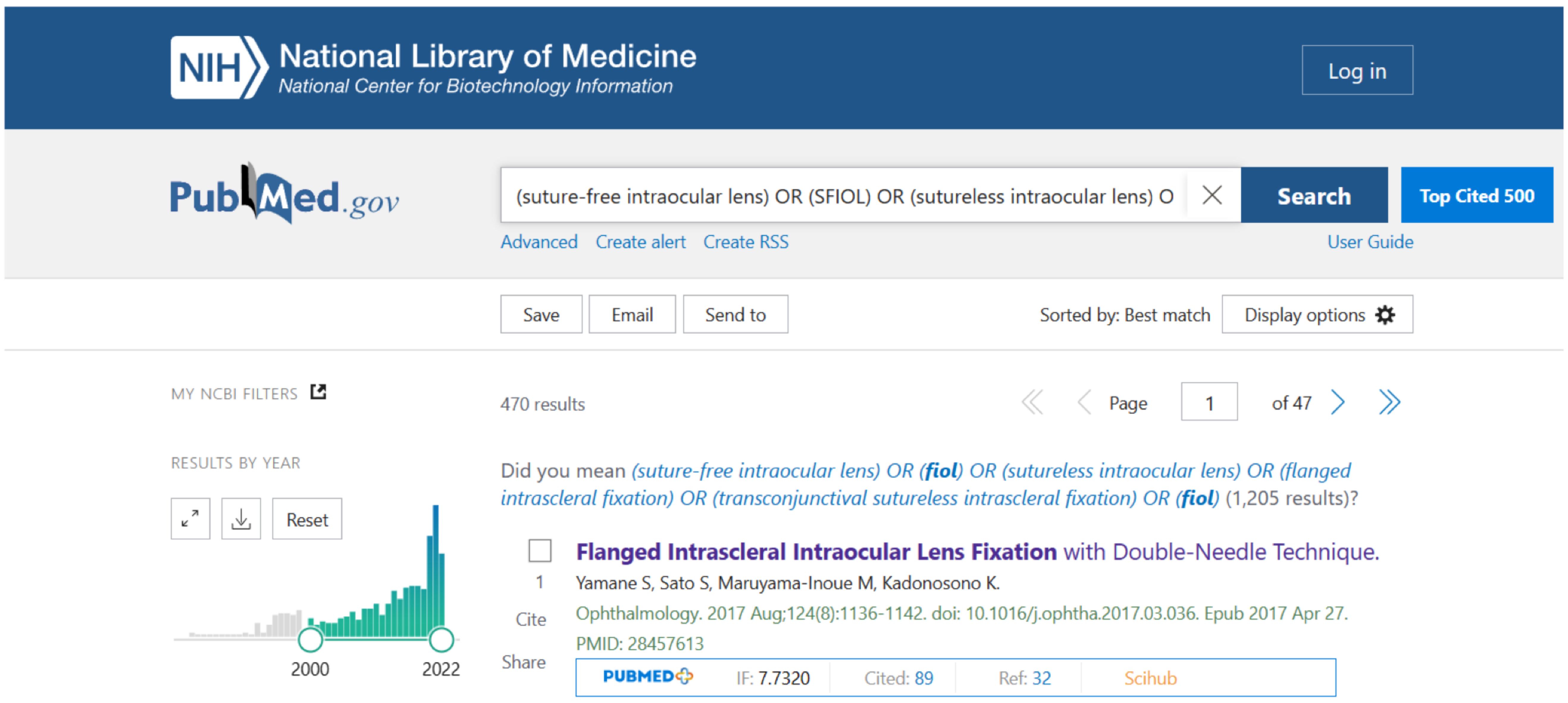

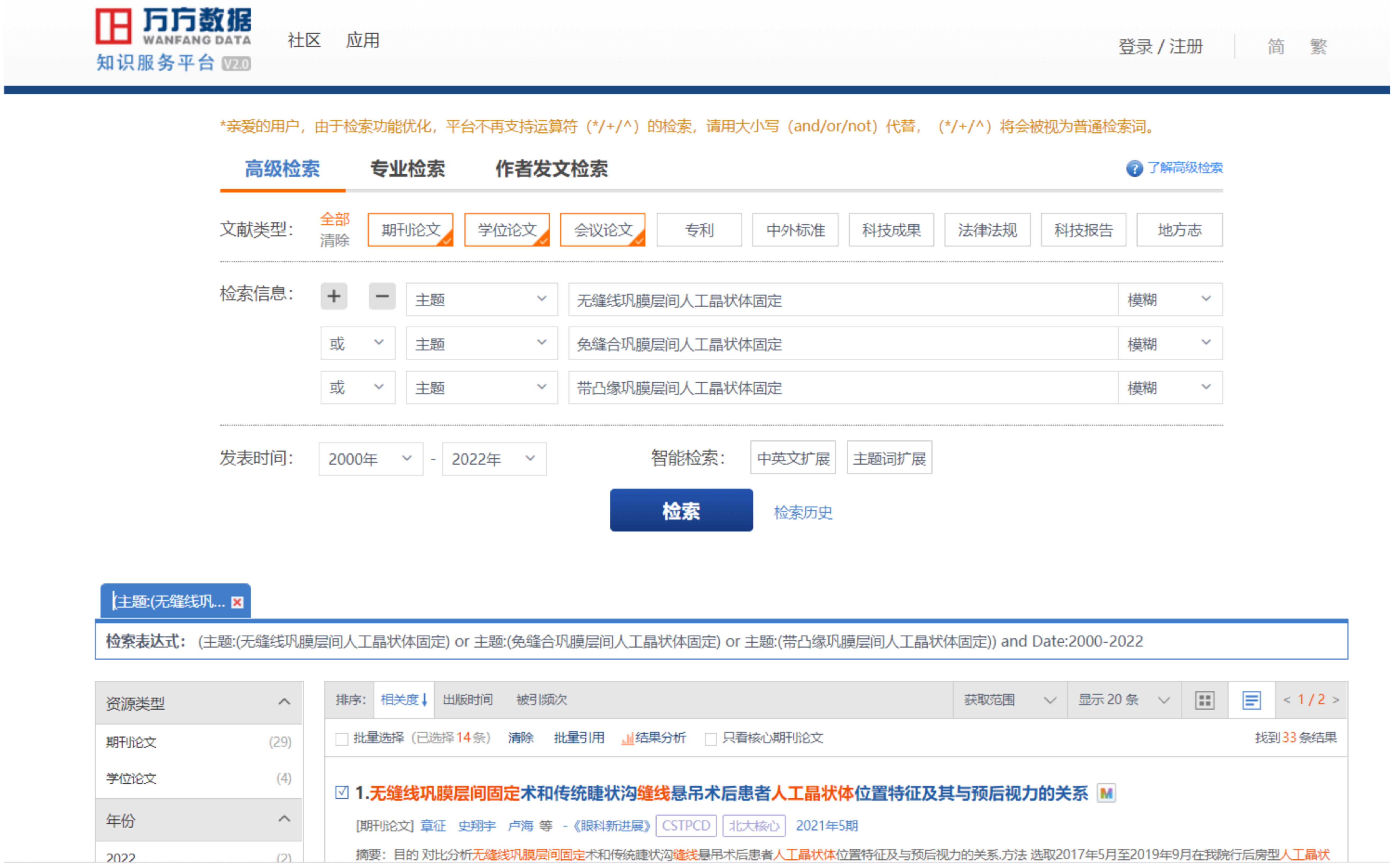

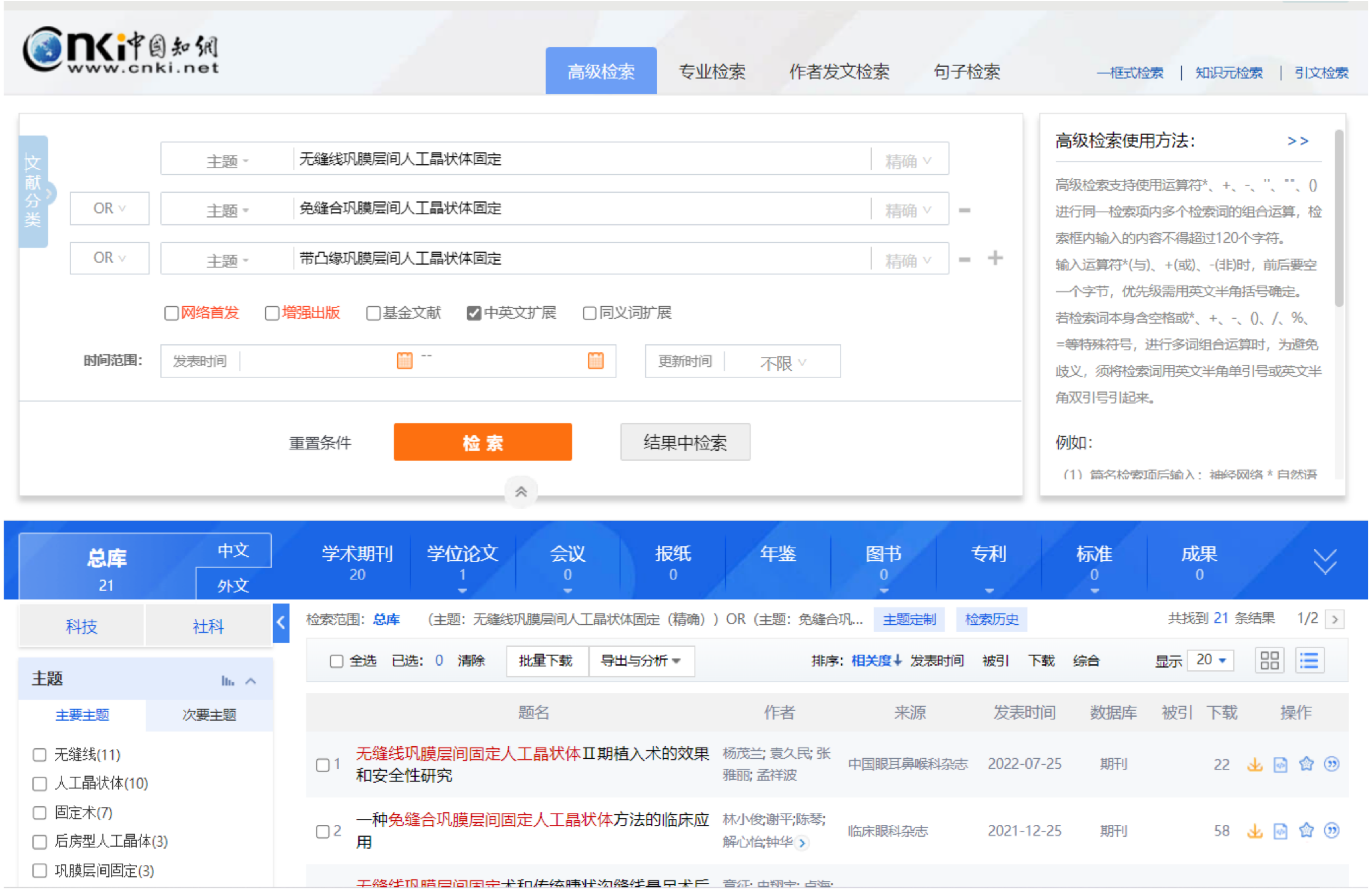
www.cnki.net
高级检索
专业检索
作者发文检索
句子检索
一框式检索 | 知识元检索 | 引文检索
文献分类
主题 无缝线巩膜层间人工晶状体固定 精确
OR 主题 免缝合巩膜层间人工晶状体固定 精确
OR 主题 带凸缘巩膜层间人工晶状体固定 精确
网络首发 增强出版 基金文献 中英文扩展 同义词扩展
时间范围: 发表时间 更新时间 不限
重置条件
检 索
结果中检索
高级检索使用方法:
高级检索支持使用运算符*、+、-、''、""、()进行同一检索项内多个检索词的组合运算，检索框内输入的内容不得超过120个字符。
输入运算符*(与)、+(或)、-(非)时，前后要空一个字节，优先级需用英文半角括号确定。
若检索词本身含空格或*、+、-、()、/、%、=等特殊符号，进行多词组合运算时，为避免歧义，须将检索词用英文半角单引号或英文半角双引号引起来。
例如:
总库 21
中文
外文
学术期刊 20
学位论文 1
会议 0
报纸 0
年鉴
图书 0
专利
标准 0
成果 0
科技
社科
主题
主要主题
次要主题
无缝线(11)
人工晶状体(10)
固定术(7)
后房型人工晶体(3)
巩膜层间固定(3)
检索范围: 总库 (主题: 无缝线巩膜层间人工晶状体固定 (精确)) OR (主题: 免缝合巩... 主题定制 检索历史
共找到 21 条结果 1/2
全选 已选: 0 清除 批量下载 导出与分析
排序: 相关度 发表时间 被引 下载 综合
显示 20
题名 作者 来源 发表时间 数据库 被引 下载 操作
1 无缝线巩膜层间固定人工晶状体Ⅱ期植入术的效果和安全性研究 杨茂兰; 袁久民; 张雅丽; 孟祥波 中国眼耳鼻喉科杂志 2022-07-25 期刊 22
2 一种免缝合巩膜层间固定人工晶状体方法的临床应用 林小俊;谢平;陈琴;解心怡;钟华 临床眼科杂志 2021-12-25 期刊 58

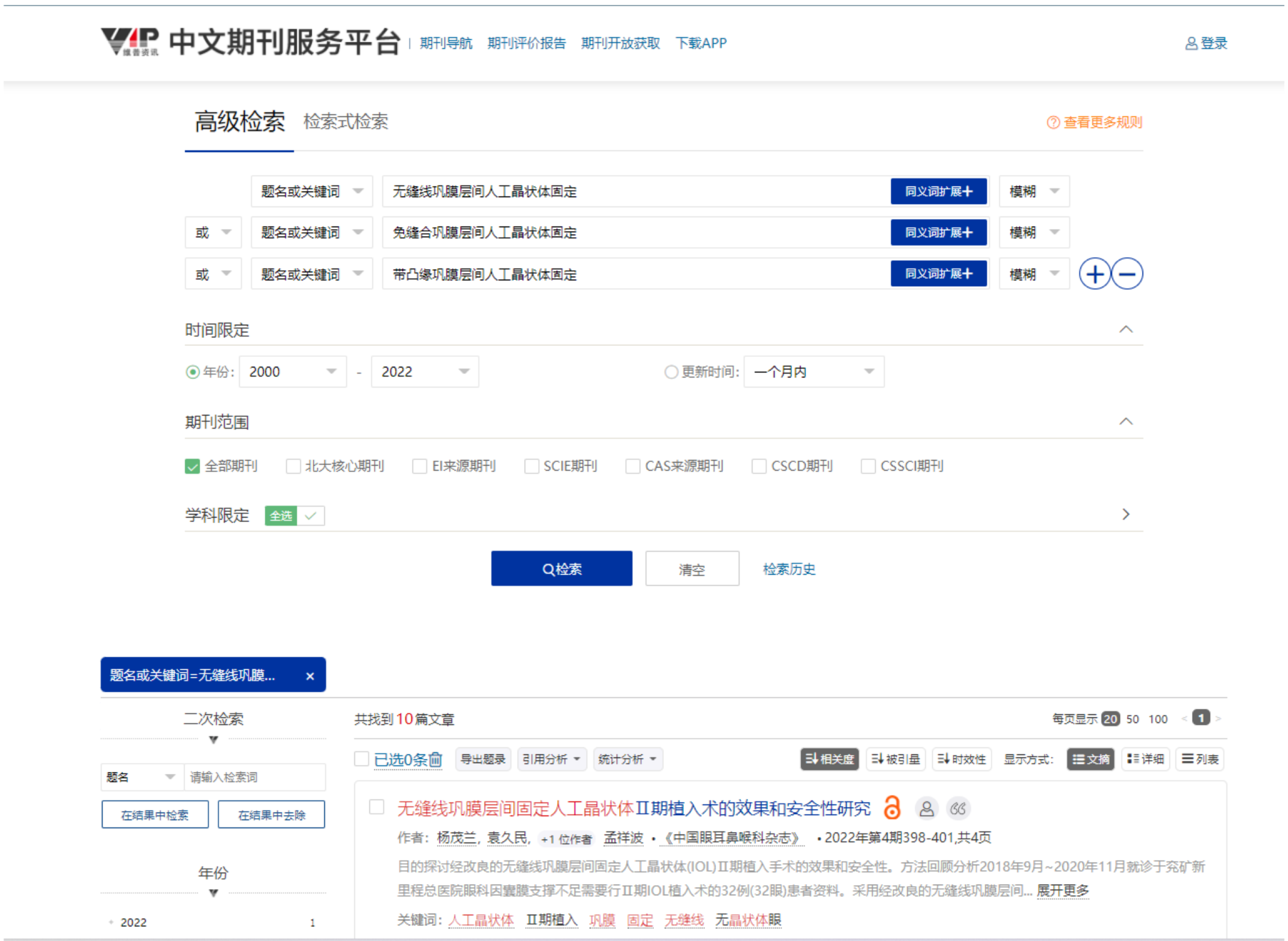
中文期刊服务平台
期刊导航
期刊评价报告
期刊开放获取
下载APP
登录
高级检索
检索式检索
查看更多规则
题名或关键词 无缝线巩膜层间人工晶状体固定 同义词扩展+ 模糊
或 题名或关键词 免缝合巩膜层间人工晶状体固定 同义词扩展+ 模糊
或 题名或关键词 带凸缘巩膜层间人工晶状体固定 同义词扩展+ 模糊
时间限定
年份: 2000 - 2022
更新时间: 一个月内
期刊范围
全部期刊
北大核心期刊
EI来源期刊
SCIE期刊
CAS来源期刊
CSCD期刊
CSSCI期刊
学科限定 全选
Q检索
清空
检索历史
题名或关键词=无缝线巩膜...
二次检索
题名 请输入检索词
在结果中检索
在结果中去除
年份
2022 1
共找到 10 篇文章
每页显示 20 50 100
已选0条
导出题录
引用分析
统计分析
相关度
被引量
时效性
显示方式:
文摘
详细
列表
无缝线巩膜层间固定人工晶状体Ⅱ期植入术的效果和安全性研究
作者: 杨茂兰, 袁久民, +1 位作者 孟祥波 • 《中国眼耳鼻喉科杂志》 • 2022年第4期398-401,共4页
目的探讨经改良的无缝线巩膜层间固定人工晶状体(IOL)Ⅱ期植入手术的效果和安全性。方法回顾分析2018年9月~2020年11月就诊于兖矿新里程总医院眼科因囊膜支撑不足需要行Ⅱ期IOL植入术的32例(32眼)患者资料。采用经改良的无缝线巩膜层间... 展开更多
关键词: 人工晶状体 Ⅱ期植入 巩膜 固定 无缝线 无晶状体眼

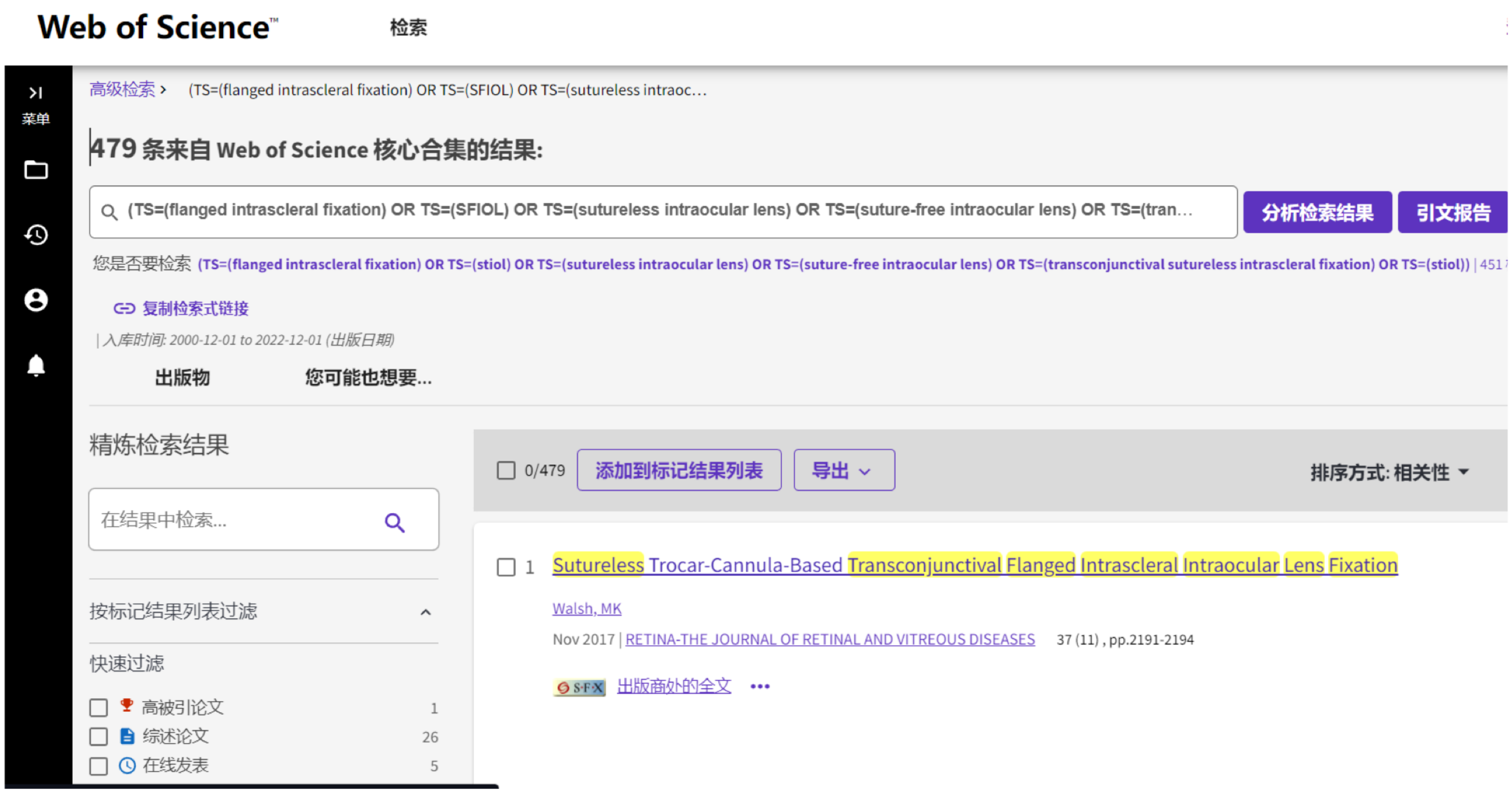
Web of Science™
检索
高级检索 › (TS=(flanged intrascleral fixation) OR TS=(SFIOL) OR TS=(sutureless intraoc…
479 条来自 Web of Science 核心合集的结果:
(TS=(flanged intrascleral fixation) OR TS=(SFIOL) OR TS=(sutureless intraocular lens) OR TS=(suture-free intraocular lens) OR TS=(tran…
分析检索结果
引文报告
您是否要检索 (TS=(flanged intrascleral fixation) OR TS=(stiol) OR TS=(sutureless intraocular lens) OR TS=(suture-free intraocular lens) OR TS=(transconjunctival sutureless intrascleral fixation) OR TS=(stiol))
复制检索式链接
入库时间: 2000-12-01 to 2022-12-01 (出版日期)
出版物
您可能也想要...
精炼检索结果
在结果中检索...
按标记结果列表过滤
快速过滤
高被引论文 1
综述论文 26
在线发表 5
0/479
添加到标记结果列表
导出
排序方式: 相关性
1 Sutureless Trocar-Cannula-Based Transconjunctival Flanged Intrascleral Intraocular Lens Fixation
Walsh, MK
Nov 2017 | RETINA-THE JOURNAL OF RETINAL AND VITREOUS DISEASES 37 (11), pp.2191-2194
出版商处的全文

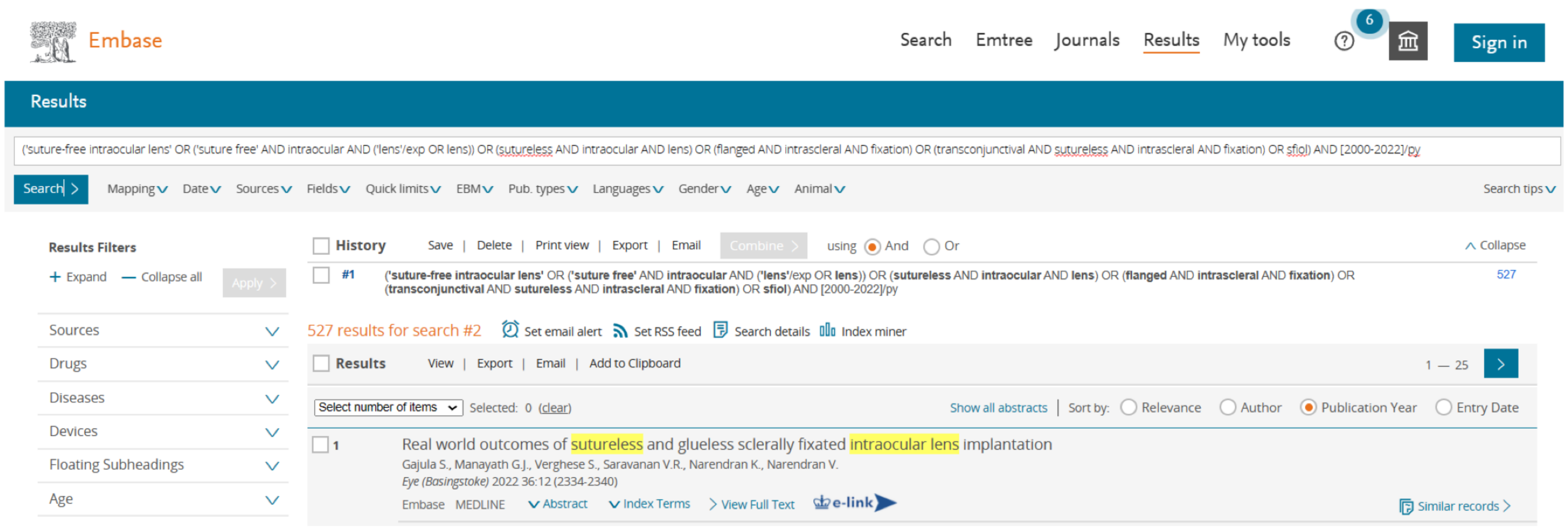
Embase
Search
Emtree
Journals
Results
My tools
Sign in
Results
('suture-free intraocular lens' OR ('suture free' AND intraocular AND ('lens'/exp OR lens)) OR (sutureless AND intraocular AND lens) OR (flanged AND intrascleral AND fixation) OR (transconjunctival AND sutureless AND intrascleral AND fixation) OR sfiol) AND [2000-2022]/py
Search
Mapping
Date
Sources
Fields
Quick limits
EBM
Pub. types
Languages
Gender
Age
Animal
Search tips
Results Filters
Expand
Collapse all
Apply
Sources
Drugs
Diseases
Devices
Floating Subheadings
Age
History
Save | Delete | Print view | Export | Email
Combine
using And Or
Collapse
#1 ('suture-free intraocular lens' OR ('suture free' AND intraocular AND ('lens'/exp OR lens)) OR (sutureless AND intraocular AND lens) OR (flanged AND intrascleral AND fixation) OR (transconjunctival AND sutureless AND intrascleral AND fixation) OR sfiol) AND [2000-2022]/py
527
527 results for search #2
Set email alert
Set RSS feed
Search details
Index miner
Results
View | Export | Email | Add to Clipboard
1 — 25
Select number of items
Selected: 0 (clear)
Show all abstracts
Sort by: Relevance Author Publication Year Entry Date
1 Real world outcomes of sutureless and glueless sclerally fixated intraocular lens implantation
Gajula S., Manayath G.J., Verghese S., Saravanan V.R., Narendran K., Narendran V.
Eye (Basingstoke) 2022 36:12 (2334-2340)
Embase MEDLINE
Abstract
Index Terms
View Full Text
e-link
Similar records

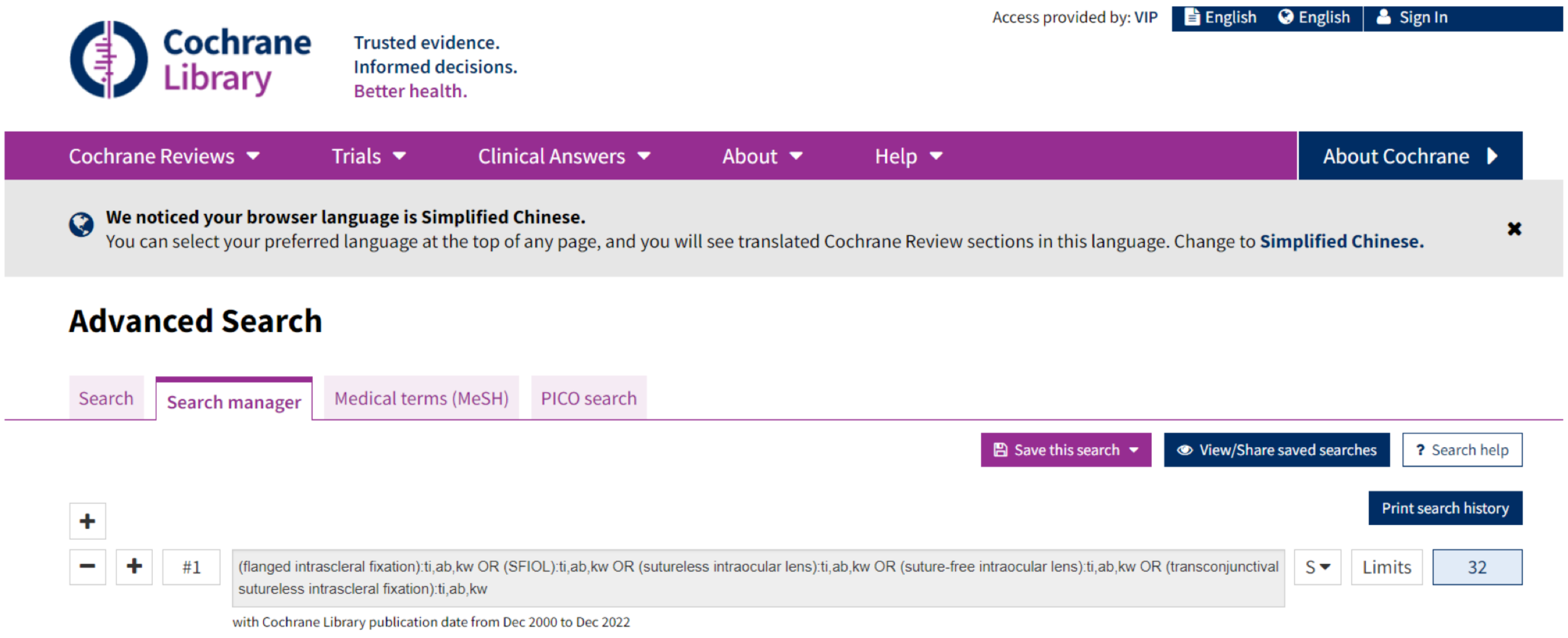
Access provided by: VIP
English
English
Sign In
Cochrane Library
Trusted evidence.
Informed decisions.
Better health.
Cochrane Reviews
Trials
Clinical Answers
About
Help
About Cochrane
We noticed your browser language is Simplified Chinese.
You can select your preferred language at the top of any page, and you will see translated Cochrane Review sections in this language. Change to Simplified Chinese.
Advanced Search
Search
Search manager
Medical terms (MeSH)
PICO search
Save this search
View/Share saved searches
Search help
Print search history
#1
(flanged intrascleral fixation):ti,ab,kw OR (SFIOL):ti,ab,kw OR (sutureless intraocular lens):ti,ab,kw OR (suture-free intraocular lens):ti,ab,kw OR (transconjunctival sutureless intrascleral fixation):ti,ab,kw
S
Limits
32
with Cochrane Library publication date from Dec 2000 to Dec 2022
