# Supplementary material for: Effect of sutureless scleral fixed intraocular lens implantation on aphakic eyes: a system review and meta-analysis
Source: BMC Ophthalmol. 2023 Dec 6;23:493. doi: 10.1186/s12886-023-03223-6 (PMC10698919; doi:10.1186/s12886-023-03223-6)
Supplement: Supplementary file 3 — Additional file 3. PRISMA 2020 flow diagram. [file 12886_2023_3223_MOESM3_ESM.pdf]

PRISMA 2020 flow diagram for new systematic reviews which included searches of databases and registers only

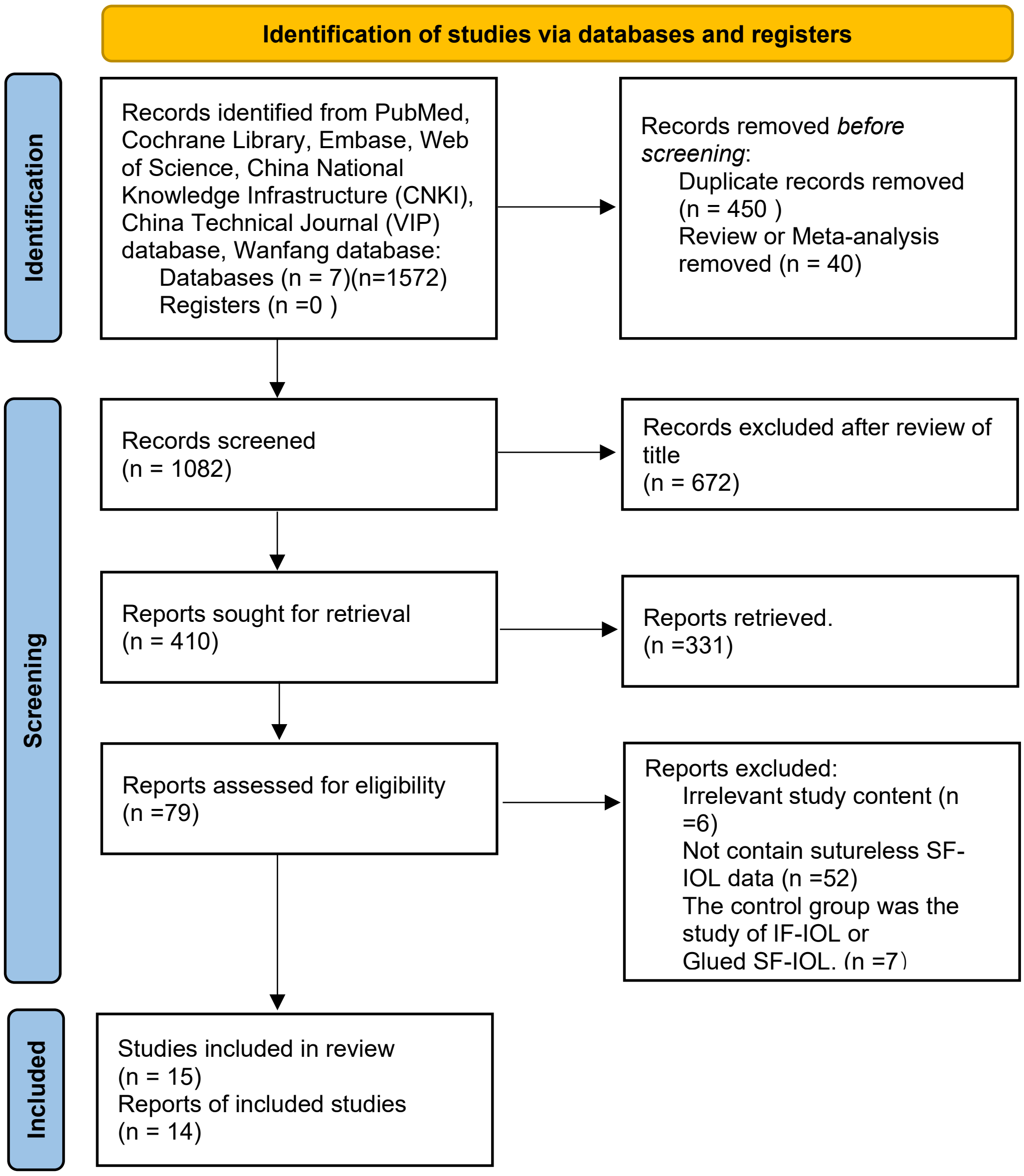

*Consider, if feasible to do so, reporting the number of records identified from each database or register searched (rather than the total number across all databases/registers).

**If automation tools were used, indicate how many records were excluded by a human and how many were excluded by automation tools.

From: Page MJ, McKenzie JE, Bossuyt PM, Boutron I, Hoffmann TC, Mulrow CD, et al. The PRISMA 2020 statement: an updated guideline for reporting systematic reviews. BMJ 2021;372:n71. doi: 10.1136/bmj.n71

For more information, visit: http://www.prisma-statement.org/
